# Upgrading CO_2_ to sustainable aromatics via perovskite-mediated tandem catalysis

**DOI:** 10.1038/s41467-024-47270-z

**Published:** 2024-04-08

**Authors:** Guo Tian, Zhengwen Li, Chenxi Zhang, Xinyan Liu, Xiaoyu Fan, Kui Shen, Haibin Meng, Ning Wang, Hao Xiong, Mingyu Zhao, Xiaoyu Liang, Liqiang Luo, Lan Zhang, Binhang Yan, Xiao Chen, Hong-Jie Peng, Fei Wei

**Affiliations:** 1 Beijing Key Laboratory of Green Chemical Reaction Engineering and Technology, Department of Chemical Engineering, Tsinghua University, 100084 Beijing, China; 2 Ordos Laboratory, Ordos, Inner Mongolia, 017010 China; 3 Institute for Carbon Neutrality, Tsinghua University, 100084 Beijing, China; 4 Institute of Fundamental and Frontier Sciences, University of Electronic Science and Technology of China, Chengdu, 611731 Sichuan China; 5 Key Laboratory of Fuel Cell Technology of Guangdong Province, School of Chemistry and Chemical Engineering, South China University of Technology, Guangzhou, 510640 China; 6 College of Chemistry, Taiyuan University of Technology, Taiyuan, 030024 China; 7 Faculty of Environment and Life, Beijing University of Technology, 100124 Beijing, China

**Keywords:** Heterogeneous catalysis, Chemical engineering, Density functional theory

## Abstract

The directional transformation of carbon dioxide (CO_2_) with renewable hydrogen into specific carbon-heavy products (C_6+_) of high value presents a sustainable route for net-zero chemical manufacture. However, it is still challenging to simultaneously achieve high activity and selectivity due to the unbalanced CO_2_ hydrogenation and C–C coupling rates on complementary active sites in a bifunctional catalyst, thus causing unexpected secondary reaction. Here we report LaFeO_3_ perovskite-mediated directional tandem conversion of CO_2_ towards heavy aromatics with high CO_2_ conversion (> 60%), exceptional aromatics selectivity among hydrocarbons (> 85%), and no obvious deactivation for 1000 hours. This is enabled by disentangling the CO_2_ hydrogenation domain from the C-C coupling domain in the tandem system for Iron-based catalyst. Unlike other active Fe oxides showing wide hydrocarbon product distribution due to carbide formation, LaFeO_3_ by design is endowed with superior resistance to carburization, therefore inhibiting uncontrolled C–C coupling on oxide and isolating aromatics formation in the zeolite. In-situ spectroscopic evidence and theoretical calculations reveal an oxygenate-rich surface chemistry of LaFeO_3_, that easily escape from the oxide surface for further precise C–C coupling inside zeolites, thus steering CO_2_-HCOOH/H_2_CO-Aromatics reaction pathway to enable a high yield of aromatics.

## Abstract (web summary)

The transformation of CO2 with renewable hydrogen into high-value products presents a sustainable route for net-zero chemical manufacture. Here the authors introduce a LaFeO3 perovskite-mediated tandem conversion of CO2, achieving remarkable performance by separating the CO2 hydrogenation and C-C coupling domains in the catalyst system.

## Introduction

Carbon dioxide (CO_2_) is the most well-known greenhouse gas leading to global warming and a range of climate and environmental issues^1,2^. To mitigate such an anthropogenic climate change, there exists an urgent need to develop solutions to CO_2_ emission. Utilizing waste CO_2_ as a feedstock and transforming it to value-added chemicals with renewable energy are therefore very promising, with both aspects of recycling CO_2_ and alleviating society’s dependence on fossil resources^3,4^. Among various CO_2_ utilization strategies, thermocatalytic CO_2_ conversion that leverages the reducing power of renewable hydrogen (H_2_) possesses a unique capability of producing complex molecules such as long-chain paraffins and heavy aromatics^5–8^. From a technoeconomic perspective, aromatics are ideal target products as additives in synthetic aviation fuels and platform molecules in many fine chemical applications^9^. Thus, renewable-powered directional conversion of CO_2_ to aromatics presents a sustainable and eco-friendly alternative to conventional fossil-resource-based chemical processes and thus could considerably contribute to decarbonizing the air transportation and chemical manufacture sectors.

Many efforts have been made to develop highly effective metal oxide/zeolite composite catalysts with methanol produced on the oxides, such as In_2_O_3_, ZnZrO_x_ and ZnAlO_x_ and so on, from CO/CO_2_ hydrogenation, and subsequently transform them into aromatics over the zeolite component such as H-ZSM-5^10–12^. The high aromatics selectivity stems from the spatially separated active domains and process in tandem; while CO_2_ and H_2_ are activated on the oxide as the hydrogenation domain, generating mainly hydrogenated C_1_ species^5,13–15^, the zeolite domain traps these species in its confined acidic pores and catalyzes the subsequent complicated transformations toward aromatics^16,17^. The selectivity can be easily regulated by tuning the structure and acidicity of zeolites. Nevertheless, the typical CO_2_ conversion in such catalytic processes is unsatisfactory (around 10–30%), which is mainly limited by the sluggish hydrogenation reactions on commonly used Zn/spinel-based oxides in the tandem processes^5,18^. Moreover, the migration of Zn/In species from metal oxide to zeolite often results in a shortened lifespan for the catalyst. These challenges underscore a critical need for innovative approaches that not only enhance CO_2_ conversion efficiency but also address the stability and longevity of the catalytic system.

On the contrary, Fe-based catalysts have been widely reported with superior activity for CO/CO_2_ hydrogenation to aromatics^19,20^; but in general, also suffer from undesirable selectivity when combined with H-ZSM-5. This process was also called enhanced Fischer–Tropsch route. To be more specific, light olefins (C_2-4_^=^) were produced over iron-based oxides domain with a selectivity at the range of ~40–65%^21–23^. Such intermediates diffuse into zeolite to open C–C coupling and cyclization reaction to produce aromatics^24,25^. However, this composite route encompasses a complex reaction network: CO_2_ hydrogenation and uncontrolled C–C coupling occur on the iron oxide, while further C–C coupling takes place inside the zeolite. This complexity often results in mismatched C–C coupling rates between oxide and zeolite and unintended isomerization of olefins within the zeolite channels. As a result, Fe oxides have seldom achieved satisfactory selectivity for aromatics among hydrocarbons (<70%)^16,26,27^ in this composite catalyzed tandem conversion of CO_2_. While extremely precise control strategies like the introduction of an appropriate amount of alkali metal, as illustrated by Sun et al.^28^, have shown to improve selectivity, they introduce a new challenge: alkali metals tend to migrate to the zeolite, diminishing catalytic performance over time^29^. To circumvent these issues, disentangling the CO_2_ hydrogenation domain from the C–C coupling domain in the tandem system may be another potential strategy for Iron-based catalyst. In other words, by maintaining robust hydrogenation activity while tempering the C–C coupling propensity on the iron oxide side, may offer great potential for highly efficient directional tandem conversion of CO_2_ to aromatics^28,30,31^.

Building on the acknowledgments above, we present a novel composite iron-oxide/zeolite system featuring LaFeO_3_ perovskite, distinctively characterized by a well-crafted [FeO_6_] octahedral framework and Fe-3d band structures, offering superior stability and exceptional resistance to carburization. This stands in stark contrast to traditional iron-based catalysts, which generally exhibit high C_2+_ selectivity. Our system uniquely suppresses Fe carbide formation, ensuring that CO_2_ hydrogenation on LaFeO_3_ yields primarily crucial C_1_ products (>90% S(CH_4_)), while C–C coupling predominantly takes place within the H-ZSM-5 zeolite, utilizing desorbed oxygenates as precursors. This synergy between LaFeO_3_ perovskite and H-ZSM-5 leads to unparalleled control over hydrocarbon selectivity, with total aromatics exceeding 85% and CO selectivity <10%—a milestone yet to be achieved by other Fe-based catalysts. Furthermore, it propels the CO_2_ conversion rate to over 60%, significantly surpassing the conventional 10–30% observed with non-Fe oxides. Notably, the LaFeO_3_/H-ZSM-5 catalyst also demonstrates extraordinary durability, maintaining performance for over 1000 h with the decay rate of aromatics yield markedly lower than that of other reported composite systems. The successful implementation of LaFeO_3_ in tandem CO_2_ conversion not only underscores the vast potential of active-metal-based perovskites in industrial CO_2_ recycling but also heralds a new era of perovskite-mediated tandem catalysis. This breakthrough provides a sustainable and scalable pathway for extensive CO_2_ utilization and chemical synthesis, paving the way for a decarbonized chemical industry.

## Results and discussion

### The design and structure of LaFeO_3_ perovskite

**Fig. 1:**
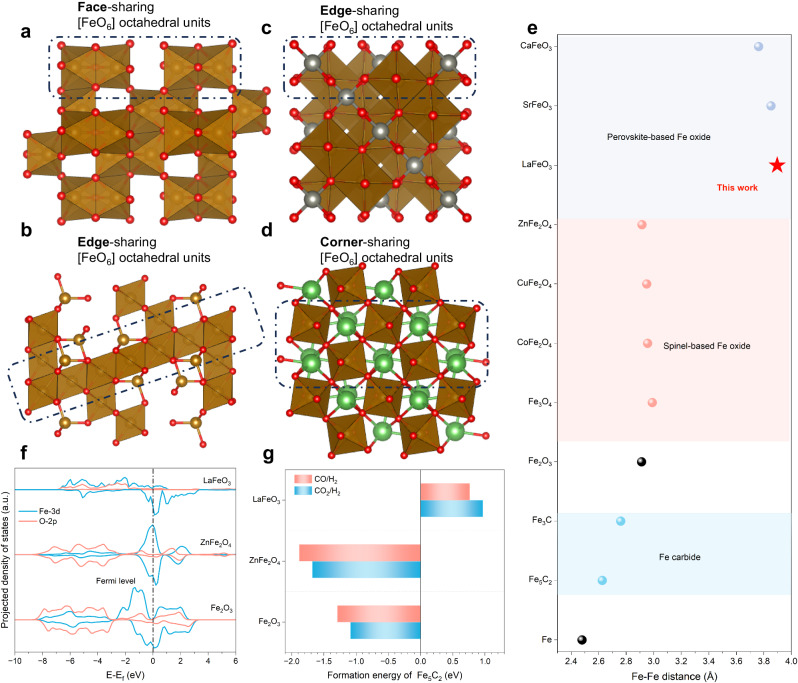
The design of LaFeO_3_ perovskite as a carburization-resistant Fe-based oxide. Atomic structural model of (**a**) Fe_2_O_3_, (**b**) Fe_3_O_4_, (**c**) ZnFe_2_O_4_, and (**d**) LaFeO_3_, showing different connection modes of [FeO_6_] octahedra. Spheres in red, brown, silver, and green represent O, Fe, Zn, and La atoms, respectively. **e** Fe–Fe distances of Fe, Fe carbides, and various Fe oxides. **f** Projected density of states (PDOSs) of Fe_2_O_3_, LaFeO_3_, and ZnFe_2_O_4_. **g** Calculated formation energies of per mole Fe_5_C_2_ through the reduction of Fe_2_O_3_, ZnFe_2_O_4_ and LaFeO_3_ by typical reactive atmospheres.

Perovskite oxides in a general formula of ABO_3_ have a framework of corner-sharing [BO_6_] octahedra with large A cations filled in the interspace^32,33^. In such a structure, Fe prefers to occupy the B site and thus enables the construction of a corner-sharing [FeO_6_] framework in Fe-based perovskites, which is quite different from the face- or edge-sharing frameworks in general Fe oxides (e.g., Fe_2_O_3_, Fe_3_O_4_) and AFe_2_O_4_-type spinels (Fig. 1a–d). Such a topological difference results in distinct Fe–Fe distances, which are expected to affect the migration of Fe if the oxide was to be carburized (Fig. 1e). Among various Fe oxides, Fe_2_O_3_ has the most compact [FeO_6_] connection and thus the shortest Fe–Fe distance of 2.92 Å; AFe_2_O_4_-type spinels or inverse-spinel Fe_3_O_4_ mainly contain edge-sharing [FeO_6_] octahedra, displaying intermediate Fe–Fe distances in the range of 2.94–3.10 Å; Fe-based perovskites normally possess much longer Fe–Fe distances of >3.7 Å. During the phase transition from Fe oxides to Fe carbides, the Fe–Fe distance has to decrease to around 2.6–2.8 Å. Therefore, the required migration distance of Fe during the phase transition is notably elongated for Fe-based perovskites. Meanwhile, the A-site cation serves as pillars pinning in the perovskite [FeO_6_] framework. We anticipate that both structural aspects of perovskite by design could lead to suppressed Fe migration during possible carburization.

As a proof of the concept, LaFeO_3_ is selected because La is a rare earth metal with the largest ion radius^34,35^, which gives rise to a remarkably long Fe–Fe distance >3.90 Å, and La has unique empty 4f orbitals that could further regulate the stability of [FeO_6_] framework through a crystal field effect. To elucidate such effect, density functional theory (DFT) calculations were performed to analyze the PDOSs of Fe_2_O_3_, ZnFe_2_O_4_, and LaFeO_3_ (Fig. 1f). It is unveiled that the incorporation of La into the corner-sharing [FeO_6_] matrix substantially reduces the number of Fe-3d states near the Fermi level when compared to Fe_2_O_3_. An opposite trend in Fe-3d states is shown for spinel ZnFe_2_O_4_. While the formation of Fe_5_C_2_ firstly involves the interaction between Fe states near the Fermi level and carbon states^21,36^, the reduction in Fe-3d states near the Fermi level can lead to significantly more energies required to form Fe_5_C_2_ and thus suppressed carburization of LaFeO_3_. Calculated formation energies of Fe_5_C_2_ further confirm the higher stability of LaFeO_3_ than Fe_2_O_3_ and ZnFe_2_O_4_ under typical reductive atmospheres consisting of CO_2_/H_2_ or CO/H_2_ (Fig. 1g). Under both atmospheres, formation energies of Fe_5_C_2_ (per mole) from Fe_2_O_3_ and ZnFe_2_O_4_ are negative, showing a strong tendency to form Fe carbides. In a sharp contrast, formation energies of Fe_5_C_2_ from LaFeO_3_ are more positive by 2.06 and 2.64 eV than Fe_2_O_3_ and ZnFe_2_O_4_, respectively. The theoretical results provide thermodynamic insights into the superior carburization resistance of LaFeO_3_, which originates from the reduced activity of Fe through electronic modulation of La.

To realize the above design, phase-pure particulate LaFeO_3_ catalyst with a size of 10–25 nm was synthesized by a microemulsion method and its ordered perovskite structure (PDF-#37-1493) was resolved by high-angle annular dark field and integrated differential phase contrast scanning transmission electron microscopy (HAADF-STEM & iDPC-STEM) and X-ray diffraction (XRD) (Supplementary Fig. 1). The distortion of ideal cubic perovskite structure is also resolved in LaFeO_3_, which is indicated by [FeO_6_] tilting through iDPC-STEM observation (Supplementary Fig. 2). The [FeO_6_] tilting is in order to accommodate the interspace large-size La cations. Hematite-structured (PDF-#33-0664) Fe_2_O_3_ and spinel-structured (PDF-#22-1012) ZnFe_2_O_4_ catalysts were obtained as control samples (Supplementary Figs. 3 and 4). All samples are shown as nanoparticles with a size ranging from 10–30 nm.

### Catalytic performance of CO_2_ conversion on different Fe catalyst

**Fig. 2:**
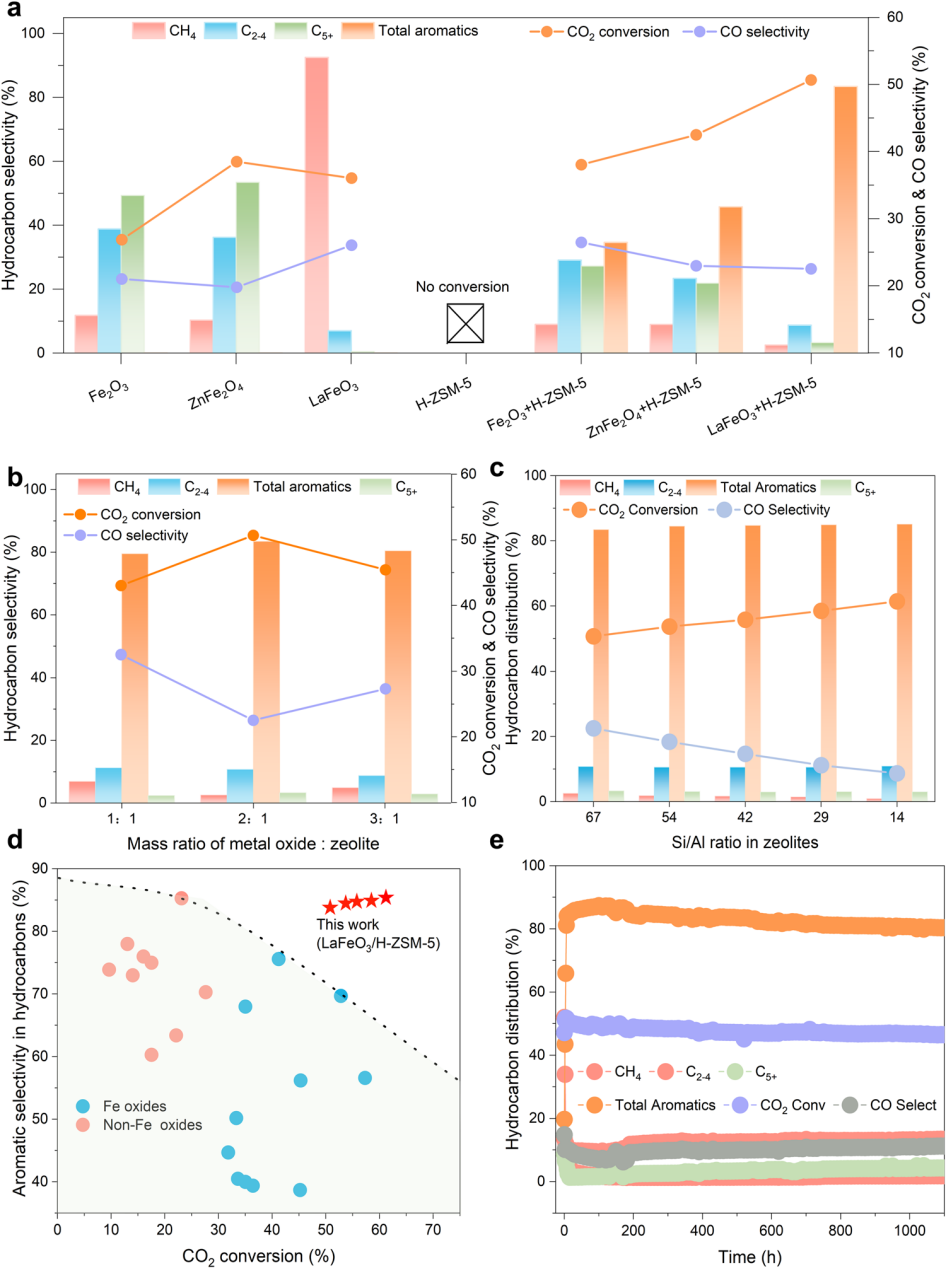
Catalytic performance of CO_2_ conversion. **a** The selectivity in hydrocarbons, CO_2_ conversion, CO selectivity of pristine oxide catalysts (left panel: Fe_2_O_3_, ZnFe_2_O_4_, and LaFeO_3_), solely H-ZSM-5 (67) zeolite, and composite tandem catalytic systems (right panel: Fe_2_O_3_/H-ZSM-5 (67), ZnFe_2_O_4_/H-ZSM-5 (67), and LaFeO_3_/H-ZSM-5 (67)) The mass ratio of oxide to H-ZSM-5 is 2:1. **b** Catalytic evaluation of LaFeO_3_/H-ZSM-5 (67) with different mass ratios. **c** Catalytic performance over LaFeO_3_/H-ZSM-5 with different Si/Al ratio. **d** Catalytic performance comparison between LaFeO_3_/H-ZSM-5 (red star) and reported non-Fe (pink cycle) and Fe (blue cycle) oxide catalysts, regarding aromatics selectivity in hydrocarbons and CO_2_ conversion. The black dashed line is a guide for the eye. The data shown in (**a**–**d**) was obtained under 3.0 MPa (H_2_:CO_2_ = 3:1) with a space velocity of 1000 ml h^−1^ g_cat_^−1^ at 350 °C. **e** Catalytic stability evaluation of LaFeO_3_ + H-ZSM-5 (14) catalyst with a more rigorous reaction condition with a space velocity of 6000 ml h^−1^ g_cat_^−1^ at 350 °C, 3.0 MPa, and CO_2_/H_2_ = 1:6.

The above three catalysts were firstly assessed under CO_2_ hydrogenation conditions (CO_2_: H_2_ = 1: 3, 350 °C, 3.0 MPa). As depicted in Fig. 2a, both Fe_2_O_3_ and ZnFe_2_O_4_ predominantly yield hydrocarbons in the C_2–4_ and C_5+_ ranges (total selectivity >87%). Such hydrocarbon distribution aligns with ASF model, corresponding to a chain growth probability (*α*) exceeding 0.7 (Supplementary Fig. 5). In contrast, LaFeO_3_ yields methane (CH_4_) as the primary hydrocarbon product (92.5%), with a substantially reduced *α* of 0.09. Since the spent Fe_2_O_3_ and ZnFe_2_O_4_ oxides suffer from notable phase transition to Fe carbides, which have been proved as excellent catalysts for C–C coupling in Fischer–Tropsch synthesis (FTS) (Supplementary Figs. 6 and 7), while the spent LaFeO_3_ does not (Supplementary Fig. 8), the notably suppressed C–C coupling on perovskite LaFeO_3_ is ascribed to its high resistance to carburization. Overall, the above results reveal the great potential of LaFeO_3_ as an isolated catalytic domain that only accounts for CO_2_ hydrogenation in tandem catalysis.

Upon integration with H-ZSM-5 (Supplementary Fig. 9), an esteemed aluminosilicate zeolite able to catalyze C–C coupling and hydrocarbon isomerization but unable to catalyze CO_2_ hydrogenation alone, marked shifts in product profiles were observed (Fig. 2a). Both Fe_2_O_3_/H-ZSM-5 and ZnFe_2_O_4_/H-ZSM-5 display increases in CO_2_ conversion ratio (38.1% and 42.5%) and aromatics selectivity (34.6% and 45.8%), which are, however, less profound than LaFeO_3_/H-ZSM-5 (Fig. 2a). C_2–4_ and C_5+_ paraffins are major hydrocarbon by-products in the two composite systems (Supplementary Fig. 10). The competition between the two C–C coupling pathways on in situ derived Fe carbides and H-ZSM-5, respectively, accounts for such broad distribution of multi-carbon products. While for LaFeO_3_/H-ZSM-5, the total aromatics selectivity in all hydrocarbons reaches 83.8% while tri-methylbenzene and tetra-methylbenzene, both of which are desirable high-calorific-value additives in aviation fuels, comprise almost 80% of total aromatics (Fig. 2a and Supplementary Fig. 10). In addition, the CO_2_ conversion ratio shows a sharp increase from 36.1% to 50.8%. This implies that H-ZSM-5 can efficiently utilize certain intermediates along the sluggish CO_2_ hydrogenation pathways on pristine LaFeO_3_. It suggests that aromatization of such intermediates in the acidic pores of H-ZSM-5 might be faster than deep hydrogenation toward CH_4_ on oxide surfaces^37,38^. The effect of LaFeO_3_: H-ZSM-5 mass ratio was further investigated (Fig. 2b). Adopting a moderate mass ratio of 2:1 yields the optimal CO_2_ conversion and aromatics selectivity. Increasing or decreasing the mass ratio both induces unwanted catalytic performance deterioration, which is ascribed to unbalanced hydrogenation and C–C coupling rates on the complementary oxide and zeolite domains.

It has been reported that the zeolite acidic property has a strong influence with the latter C–C coupling reaction and inhibition of CO-by product^28,39^. Therefore, we also conducted LaFeO_3_/H-ZSM-5 with different Si/Al ratio (Supplementary Fig. 11). As shown in Fig. 2c, with the increasing acidic properties in zeolite, the CO-by product has been inhibited from ~22% to 9.3% and CO_2_ conversion has been increased from 50.8 to 61.4%. It can be deduced that intermediates produced over LaFeO_3_ may have two subsequent reactions. One is diffuse into zeolite for further C–C coupling to produce aromatics and another is decomposed into CO-by product when the H-ZSM-5 acidic sites are deficient.

To further demonstrate such superiority, we compare the catalytic performance of LaFeO_3_/H-ZSM-5 with other reported state-of-the-art composite catalysts for CO_2_ conversion to aromatics under similar conditions (Supplementary Table 1). As shown in Fig. 2d, LaFeO_3_/H-ZSM-5 exhibits not only at least a-fold higher CO_2_ conversion rate than non-Fe oxide catalysts but also the highest aromatics selectivity in hydrocarbon. The two aspects are attributed to highly active Fe-based sites for CO_2_ hydrogenation and the unique carburization-resistant perovskite structure by design, respectively. As a consequence, LaFeO_3_ and H-ZSM-5 jointly enable strict confinement of cascade CO_2_ hydrogenation and aromatics formation at spatially separated domains, leading to superior catalytic performance for directional tandem conversion of CO_2_ to aromatics.

LaFeO_3_/H-ZSM-5(14) was also subjected to a continuous reaction durability test at more rigorous reaction condition. It is revealed that the aromatics selectivity and CO_2_ conversion rate maintain as >80% and >47%, respectively, for over 1000 h on stream (Fig. 2e). When focusing on STY of total aromatics, which serves as an integrated index of the catalytic performance, LaFeO_3_/H-ZSM-5 exhibits an extremely low decay rate of initial STY (0.021% per hour), which corresponds to 3.4–17.6 times slower performance deterioration than reported composite catalysts for CO_2_-to-aromatics conversion (Supplementary Fig. 12). While compared to conventional Fe- or Zn-based catalysts that easily suffer from carbon deposition and/or sintering and thus rapidly deactivate^40–43^, the perovskite-structured LaFeO_3_ demonstrates a great potential for industrial applications due to the record high stability, as well as a decent STY of aromatics at high selectivity. Besides, more stability tests including conventional H-ZSM-5 with nanosized H-ZSM-5 were also carried out shown in Supplementary Fig. 13.

### Structural stability of LaFeO_3_ under reactive atmosphere

**Fig. 3:**
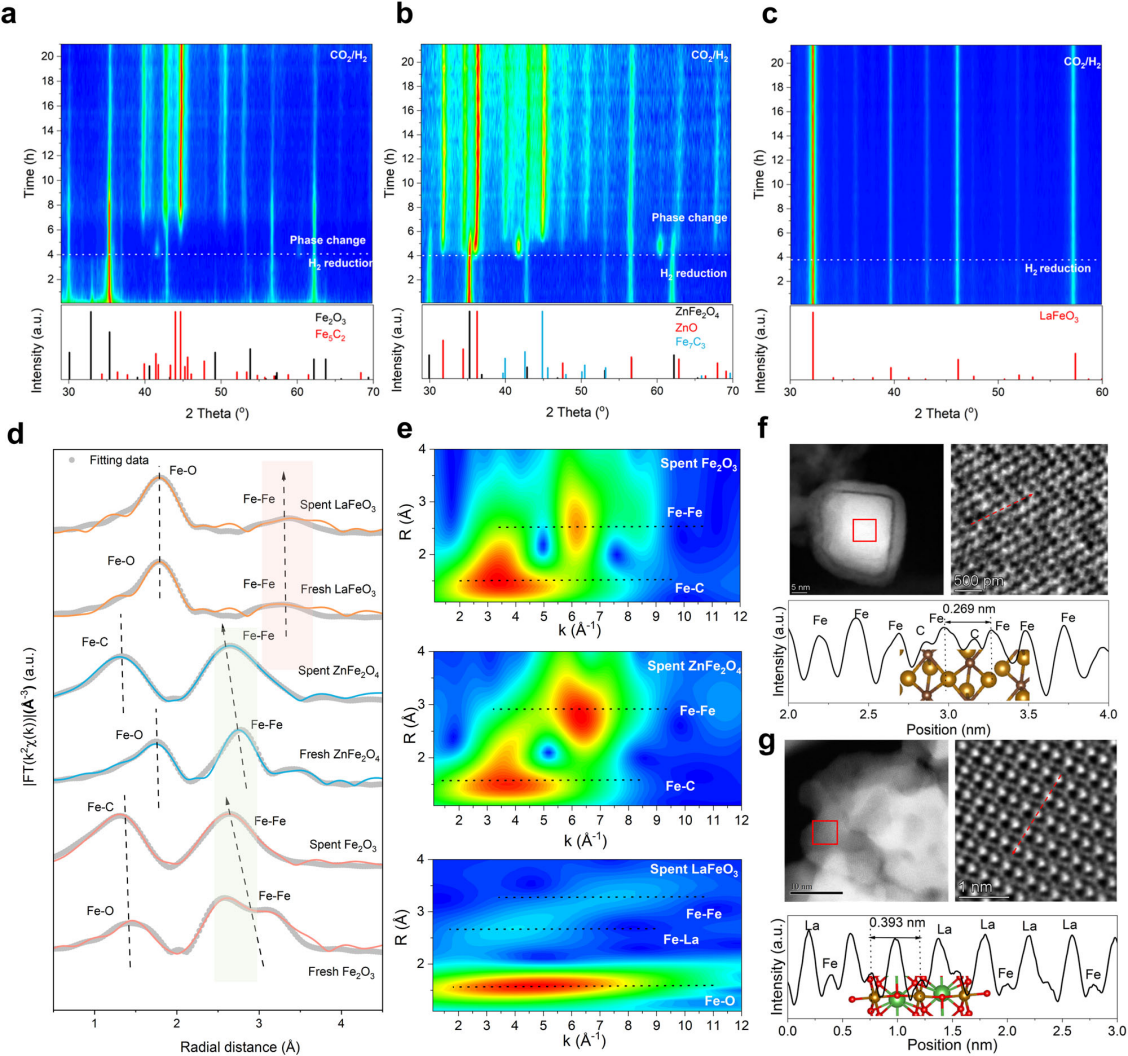
Structural evolution of different Fe catalysts under reactive atmospheres. In situ XRD patterns of (**a**) Fe_2_O_3_, (**b**) ZnFe_2_O_4_ and (**c**) LaFeO_3_ under different reductive atmosphere. Test conditions: *T* = 350 °C, *P* = 0.8 MPa and H_2_ (the first 4 h) or CO_2_/H_2_ = 1:3 (the subsequent 20 h). **d** Fe K-edge Fourier-transform extended X-ray absorption fine structures (FT-EXAFSs). **e** Fe K-edge wavelet transform (WT)-EXAFSs of spent Fe_2_O_3_, ZnFe_2_O_4_, and LaFeO_3_ catalysts. High-angle annular dark-field scanning transmission electron microscopy (STEM), integrated differential phase contrast (iDPC) STEM images, and intensity profiles of the iDPC-STEM images along imaginary line of spent (**f**) Fe_2_O_3_ and (**g**) LaFeO_3_. The insets show the atomic structure with spheres in red, black, brown, and green representing O, C, Fe, and La atoms, respectively.

The high aromatic selectivity and superior catalytic stability of LaFeO_3_ perovskite align well with the anticipation that LaFeO_3_ perovskite could be an especially different carburization-resistant catalyst from normal and spinel Fe oxides. To further obtain experimental insights into the catalyst evolution under reactive atmosphere, combinatorial in situ and ex situ spectroscopic and microscopic characterizations were performed. In situ X-ray diffraction (XRD) first reveals that Fe_2_O_3_ and ZnFe_2_O_4_ could maintain their original bulk structures upon initial H_2_ reduction but gradually transform to Fe_5_C_2_ and a mixture of ZnO and Fe_7_C_3_, respectively, once carbonaceous gas (CO_2_) being introduced into the in situ observation chamber to resemble the reactive atmosphere (Fig. 3a, b and Supplementary Fig. 14). Distinctively, the perovskite structure of LaFeO_3_ is well retained with no new crystal phases generated under either H_2_ or CO_2_/H_2_ atmospheres (Fig. 3c and Supplementary Fig. 15). In addition to the bulk structure evolution, the surface evolution was investigated by in situ X-ray photoelectron spectroscopy (XPS). Fe_2_O_3_ and ZnFe_2_O_4_ show similar patterns that a portion of Fe^3+^ is firstly reduced to lower-valence Fe^2+/+/0^ by H_2_ and then Fe^0^ is, if partially, re-oxidized to Fe^+^ in mixed CO_2/_H_2_ (Supplementary Fig. 16a, b). The emergence of Fe^+^ corresponds well with the nominal valence of Fe in its carbides. While for LaFeO_3_ perovskite Fe oxide, either reductive atmosphere only results in more unsaturated Fe^2+^ but no Fe^+^/Fe^0^ (Supplementary Fig. 16c). This is accompanied with increased amounts of oxygen vacancy (O_v_) (Supplementary Fig. 17). O_v_ might serve as the sites for CO_2_ activation of CO_2_ and subsequent hydrogenation steps^38,44^, of which the role in catalysis we will discussed in a later section.

Ex situ synchrotron X-ray characterizations were further employed to investigate the chemical status and fine local structures of spent Fe oxide catalysts. Normalized Fe K-edge X-ray absorption near-edge structures unravel the significantly reduced oxidation state of Fe in Fe_2_O_3_ and ZnFe_2_O_4_ after 24-h reaction, in line with the in situ XPS observation (Supplementary Fig. 18). In FT-EXAFS, the second-shell peak that is assigned to Fe–Fe coordination shifts to positions at shorter distances for Fe_2_O_3_ and ZnFe_2_O_4_ after 24-h reaction (Fig. 3d)^45,46^. More detailed structural information beneath the raw FT-EXAFS data was further extracted after fitting (Supplementary Table 2)., Fe–Fe coordination numbers in spent Fe_2_O_3_ and ZnFe_2_O_4_ both increase to above 7, indicating the formation of Fe carbides with more agglomerated Fe. Unlike Fe_2_O_3_ and ZnFe_2_O_4_, LaFeO_3_ exhibits marginally changed oxidation state of Fe, Fe–Fe distance (originally longer than Fe_2_O_3_ and ZnFe_2_O_4_), and Fe–Fe coordination number after the same treatment. WT-EXAFS provides additional insights into the catalyst evolution (Supplementary Fig. 19 and Fig. 3e). The Fe–Fe scattering in spent LaFeO_3_ has lower intensity than spent normal Fe_2_O_3_ and spinel ZnFe_2_O_4_, indicating a weaker Fe–Fe interaction and thus suppressed Fe migration and aggregation^47–50^.

Lastly, high-resolution electron microscopy was carried out to reveal the morphological evolution of spent Fe oxide catalysts. For the spent Fe_2_O_3_, a large quantity of Fe_x_C_y_ were detected with carbon-interstitial aggregated Fe, showing a short Fe–Fe distance of 0.269 nm (Fig. 3f); while LaFeO_3_ is shown to retain the particulate morphology with negligible particle size variation, as well as the fine perovskite structure with an alternate La and Fe arrangement (Fig. 3g). Intensity profiles of the iDPC-STEM images along imaginary line depict the preservation of long Fe–Fe distance (0.393 nm) and the existence of O_v_ (Supplementary Fig. 20). All above in situ and ex situ diffraction, spectroscopic, and microscopic results validate the superior structural stability and high carburization resistance of perovskite LaFeO_3_ in reactive atmosphere, thereby substantiating the rationality of our structural design and theoretical prediction that a long Fe–Fe distance and electronic structure modulation through La pillaring can effectively suppress Fe migration.

### Mechanistic understandings of CO_2_ conversion on LaFeO_3_. (CO_2_ to H_2_CO/H_2_CO to aromatics)

**Fig. 4:**
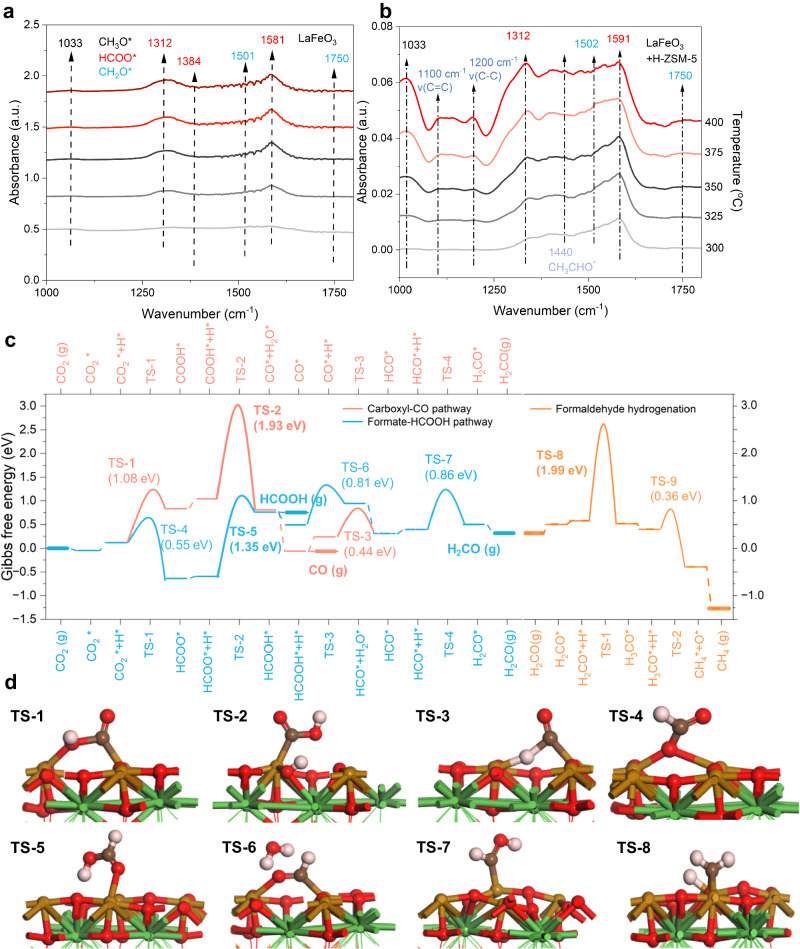
Mechanistic investigation of CO_2_ conversion on LaFeO_3_. Temperature-dependent in situ DRIFTS spectra over (**a**) LaFeO_3_ and (**b**) LaFeO_3_ + H-ZSM-5. **c** Free energy diagrams of CO_2_ hydrogenation on the surface of the LaFeO_3_ (220) surface with O_v_, showing carboxyl (red) and formate (blue) pathways toward formaldehyde and the formaldehyde hydrogenation pathway (orange) to produce CH_4_. To indicate the effect of molecular desorption, the states of gas-phase molecules are highlighted as bold horizontal lines. The competition between molecule desorption and further hydrogenation could be elucidated in the cases where gaseous HCOOH and H_2_CO are assumed to be reactants for subsequent hydrogenation. **d** TS, transition states structure over LaFeO_3_ during CO_2_ to CH_4_. Spheres in black, red, brown, and green represent C, O, Fe, and La atoms, respectively.

In addition to structural stability, the unique ability of LaFeO_3_ to selectively catalyzing CO_2_ conversion to C_1_ species, as well as the synergy between LaFeO_3_ and H-ZSM-5 in enabling high-efficiency directional CO_2_-to-aromatic conversion, is disclosed. In situ diffuse reflectance Fourier transform infrared spectroscopy (DRIFTS) was firstly employed to probe key species potentially dominating the reactions on oxide surfaces with varying temperature. On LaFeO_3_, only C_1_ oxygenated species such as formate (HCOO* at 1318 cm^−1^ and 1591 cm^−1^; the asterisk indicates the surface-adsorbed state), formaldehyde (H_2_CO* at 1151 cm^−1^), and methoxy (H_3_CO* at 1038 cm^−1^) were observed (Fig. 4a)^51–53^. Note that the intensity of the above characteristic peaks increases to maximum until temperature rises to above 350 °C (Supplementary Fig. 21). Besides, no signals related to multicarbon species are observed for solely LaFeO_3_. When combined with H-ZSM-5, the intensities of reflection bands associated with above C_1_ oxygenated species decrease by an order of magnitude (Fig. 4b), accompanied by the emergence of a prominent v(C=C), v(C–C) band at 1100, 1200 cm^−1^, respectively, implying that H-ZSM-5 has an ability to convert C_1_ oxygenated species to multicarbon products (Supplementary Figs. 22 and 23). Besides, also CH_3_CHO* species can be found at 1440 cm^−1^. The Aldol or Prins mechanism has been previously suggested to account for this ability^42,54^. For both LaFeO_3_ and LaFeO_3_/H-ZSM-5, the intensities of characteristic bands maintain unchanged after a short induction period, indicating a relatively steady state of LaFeO_3_ surfaces.

Such an oxygenate-dominated surface chemistry of LaFeO_3_ is quite distinct from that on Fe_5_C_2_ (derived from Fe_2_O_3_), a variety of C=C stretching modes (1100 cm^−1^) and C–H stretching/bending modes (1350–1600 cm^−1^) indicate the presence of various hydrocarbons (Supplementary Fig. 23). These features are typical for active metal carbide catalysts in FTS that involve CH_x_ polymerization. Thus, these observed patterns are attributed to the formation of Fe carbides. Notably, the intensities of C=C bands continuously rise with increasing reaction temperature, indicating the retention and accumulation of carbonaceous species that eventually passivate the surface. This is consistent with the rapid deactivation of normal Fe_2_O_3_ and its derived catalysts.

In light of the oxygenate-rich surface chemistry of LaFeO_3_ that mainly leads to C_1_ products, reaction energetics were investigated using DFT calculations. The LaFeO_3_ (220) surface with or without surface O_v_ was considered for simulations (Supplementary Fig. 24)^55^, and formation Gibbs free energies of relevant surface species/states, including transition states (TSs), after geometry optimization were computed (Supplementary Figs. 25–28). In general, adsorption strengths of surface species on the surface without O_v_ are notably weak, making the O_v_-free surface inert for CO_2_ hydrogenation (Supplementary Table 3). Therefore, we then focus on the surface with O_v_ to investigate the thermodynamics and kinetics of various reaction pathways.

As depicted in Fig. 4c, the CO_2_ hydrogenation firstly involves hydrogen (H*) adsorption on the O site and CO_2_ adsorption on the O_v_ near the Fe site. While two channels for CO_2_* hydrogenation open to either HCOO* or carboxyl (COOH*), reaction pathways bifurcate to two major branches, with one proceeding through CO* and the other through formic acid (HCOOH*), until the formation of formyl (HCO*). Once forming HCO*, the two branches merge again, thereafter followed by sequential hydrogenation to form H_2_CO*, H_3_CO*, and CH_4_ at the end (Fig. 4c). Both the carboxyl and formate pathways are primarily limited by the second hydrogenation step, which has to overcome an activation barrier of 1.93 eV (TS-2) and 1.35 eV (TS-5) to yield CO* + H_2_O* and HCOOH*, respectively. Therefore, the formate pathway is more kinetically accessible than the carboxyl pathway on O_v_-containing LaFeO_3_ (220), which is consistent with the strong signals of HCOO* (exclusively presented in the formate pathway) in the in situ DRIFTS measurements. A minor pathway initiating from formate, namely HCOO* hydrogenation proceeds via formaldehyde oxide (H_2_COO*), was also investigated but there presents a high barrier of 2.02 eV (Supplementary Fig. 29).

The reaction thermodynamics and kinetics rationalize several experimental observations. First, the dominant formate pathway results in a plenty of oxygenated species as probed by in situ DRIFTS. Second, the weak adsorption of HCOOH (0.75 eV) and H_2_CO (0.31 eV), as well as the high barrier for further hydrogenation of H_2_CO (1.99 eV, TS-8), dictates the disparity in CO_2_ conversion between pure LaFeO_3_ and LaFeO_3_/H-ZSM-5. On pure LaFeO_3_, the easy desorption of these species reduces their surface coverages, or equivalently, increase the free energy penalties of subsequent hydrogenation if we assume to start with HCOOH or H_2_CO gas, both of which were experimentally detected on pure LaFeO_3_ during hydrogenation reaction (Supplementary Fig. 30). While for LaFeO_3_/H-ZSM-5, the conversion of desorbed HCOOH and/or H_2_CO in the acidic pore of H-ZSM-5 is thermodynamically very favorable toward aromatics (Supplementary Table 4) and the barriers of aromatization have been demonstrated fairly low^10,56^. Therefore, the enhancement in CO_2_ conversion is realized after including H-ZSM-5 in the whole system. Third, due to the lower barrier of HCO* decomposition (backward; TS-3) than that of HCO* hydrogenation (forward, TS-7), the formation of CO through an indirect pathway (CO_2_ → HCOO* → HCOOH → HCO* → CO) becomes more viable than the direct carboxyl pathway. Such a theoretical insight may enlighten future efforts in regulating the adsorption and conversion of surface formyl to realize low CO selectivity.

**Fig. 5:**
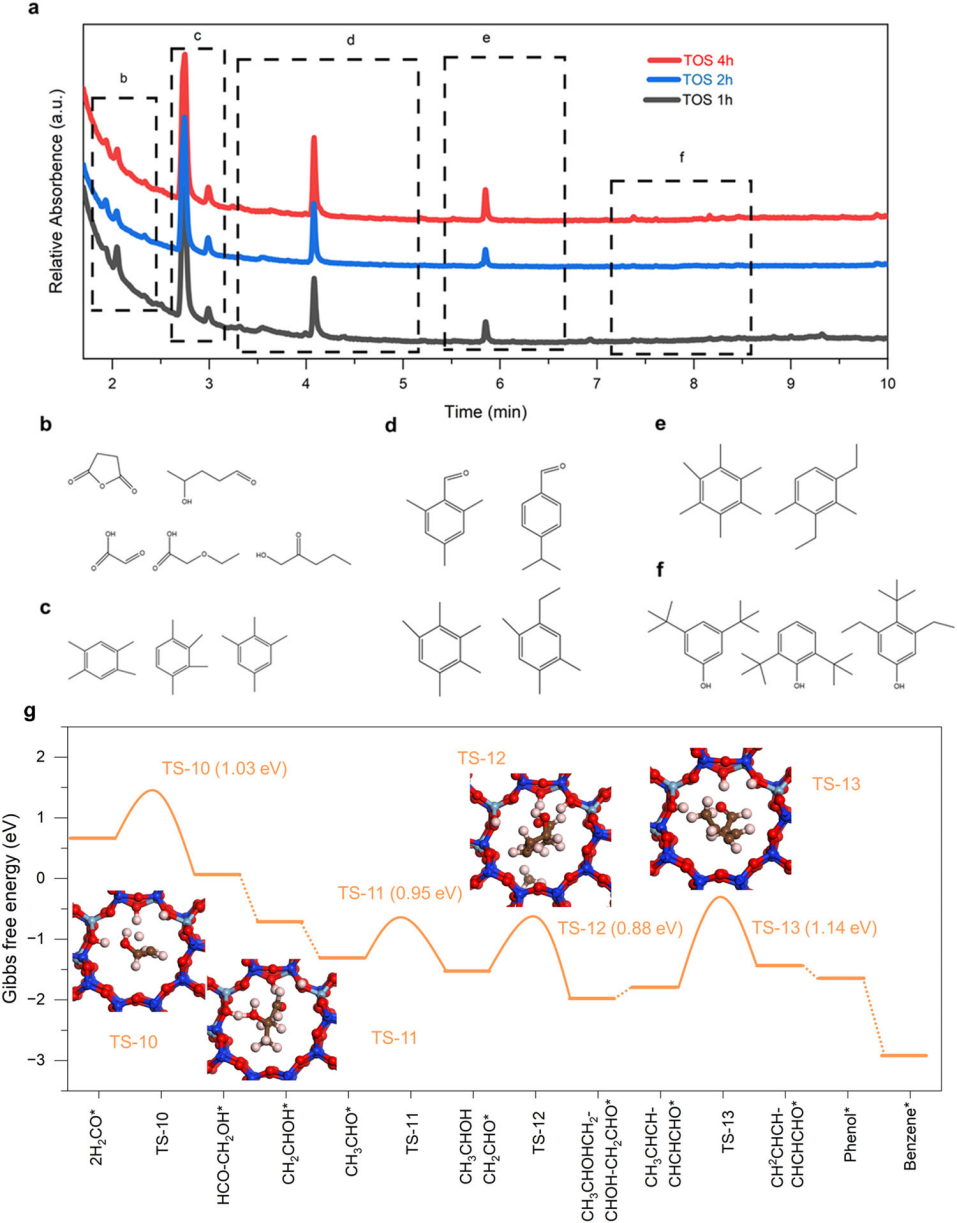
The subsequent Aldol-aromatic reaction pathway inside zeolites from H_2_CO as a key intermediate. **a** Identified carbonaceous species retained in the spent LaFeO3/H-ZSM-5(14) during different reaction time. **b**–**f** The species determined by gas chromatography-mass spectrometry. **g** H_2_CO as the key intermediates in the reaction pathway to form aromatics (aldol-aromatic) inside zeolite. Some typical transition structure during CH_2_O to aromatics over LaFeO_3_/H-ZSM-5 catalyst. Spheres in white, brown, red, light blue, and blue represent H, C, O, Al and Si, atoms, respectively.

To substantiate HCOOH/H_2_CO as pivotal intermediates in this tandem catalysis, we further explored the aldol-aromatic reaction within H-ZSM-5 (Si/Al = 15)^12,56^. One experimental procedure developed by the previous method has been carried out to detect the species inside zeolite during different time on stream (TOS). It can be found that some of aldol condensates, aromatic aldehydes and phenols-type aromatics along with multi-methylbenzene (Fig. 5a–f). The decrease of aldol condensates from 1 h to 4 h demonstrates that aldol reaction plays a significant role in the initial induction period. In addition, evidence shows that aromatic aldehydes, phenols also exhibit considerable amounts in the TOS of 1 h and 2 h in induction, indicating the feasibility for the transformation of aldol condensates into aromatic aldehydes and some phenolic oxygenates, which shows that a reaction pathway of aldol-aromatic as we speculated. Besides, the detailed reaction route was also considered by using DFT calculation inside H-ZSM-5. As delineated in Fig. 5g, h, we assumed that CH_2_O species rapidly diffused into H-ZSM-5, instigating a series of transformations into aldol condensates. Specifically, two H_2_CO molecules initially adsorb near the Al-OH site, initiating the first C–C coupling. This process results in the formation of CH_3_CHO species (also observed in Fig. 4b), which then enters the aldol reaction, facilitating further C–C coupling until long-chain six-carbon oxygenates are formed. Subsequently, these undergo cyclization and aromatization, a process influenced by the confinement and acidic properties of H-ZSM-5, ultimately leading to the formation of benzene (Supplementary Fig. 31). From a thermodynamic perspective, the energy profile within H-ZSM-5 is significantly lower than that of the C_1_ species on the LaFeO_3_ surface. This observation indicates that the zeolite effectively alters the reaction pathway from CO_2_-H_2_CO-CH_4_ to a more desirable CO_2_-H_2_CO-Aromatics sequence. Such computational insights coherently align with our experimental results, offering a robust rationale for the observed transformation pathway and underscoring the crucial role of HCOOH/H_2_CO as intermediates in this sophisticated tandem catalysis process.

**Fig. 6:**
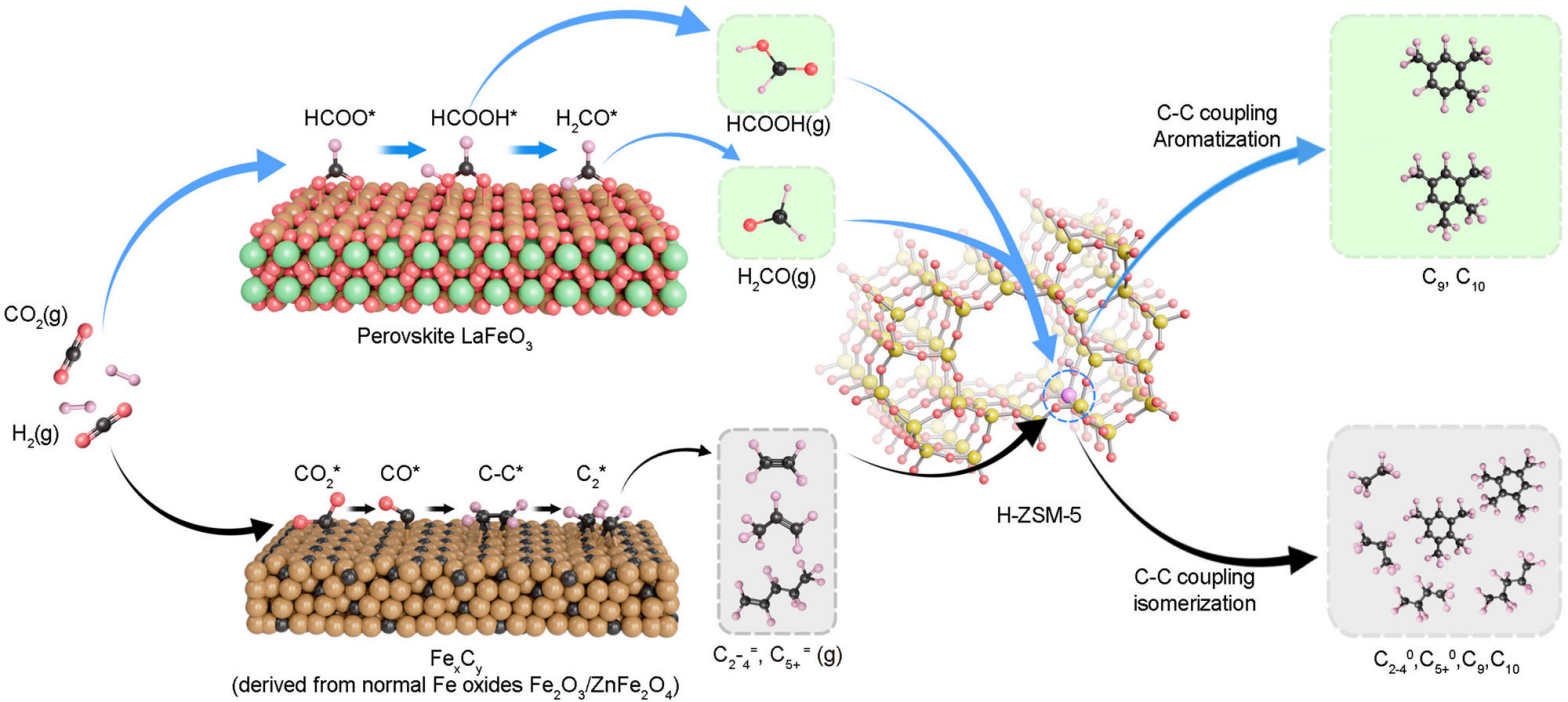
Schematic illustration of the CO_2_ conversion pathways on the bifunctional LaFeO_3_/H-ZSM-5 composite catalyst and the Fe_5_C_2_/H-ZSM-5 catalyst, in which Fe_5_C_2_ is derived from in situ transformation of conventional Fe oxide catalyst (e.g., Fe_2_O_3_ and ZnFe_2_O_4_) under the reactive atmosphere. Spheres in pink, black, red, magenta, yellow, brown, and green represent H, C, O, Al, Si, Fe, and La atoms, respectively.

Overall, the experimental and theoretical results validate our rational design of LaFeO_3_ perovskite as a highly active and carburization-resistant oxide domain for composite-catalyzed directional tandem conversion of CO_2_ to aromatics (Fig. 6). The high aromatics selectivity is attributed to deliberately decoupled formation of C_1_ oxygenates such as HCOOH and H_2_CO on LaFeO_3_ and formation of aromatics in the acidic zeolite, respectively. Consequently, strict control of hydrocarbon selectivity toward value-added heavy aromatics is realized, endowing this CO_2_ utilization route excellent process economy. While for normal or spinel Fe oxides such as Fe_2_O_3_ or ZnFe_2_O_4_, phase transformation to Fe carbides is inevitable and a sequential FTS mechanism has been proposed, giving rise to broad product distribution complying the ASF distribution. The unsatisfactory selectivity will substantially increase the cost of product separation.

In this study, we present combinatorial experimental and theoretical investigations that rationalize the design of LaFeO_3_ perovskite to mediate directional tandem conversion of CO_2_ with high conversion efficiency (>60%), exceptional aromatics selectivity (>85%), and catalytic stability for 1000 h. The coupling of active and carburization-resistant perovskite with zeolite renders strictly decoupling of CO_2_ hydrogenation and C–C coupling, offering a radically different approach from conventional industrial routes for aromatics synthesis and showcasing a new design principle of the oxide domain in tandem catalysis. In a broader context, given the large variety and fruitful chemistries of perovskites and zeolites, our strategy endows sustainable CO_2_ utilization many opportunities by further improving the directional CO_2_-to-aromatics conversion or engineering novel tandem processes to yield other value-added products. Since renewable electricity and green hydrogen are both at increasingly low cost, perovskite-mediated directional tandem conversion of CO_2_ is ready for renewable-driven greenhouse gas upcycling and chemical industry decarbonizing.

## Methods

### Synthesis of Fe_2_O_3_ oxide

Fe_2_O_3_ was synthesized via co-precipitation method. Typically, 16.16 g of Fe(NO_3_)_3_·9H_2_O was added to 45 ml of deionized water (DI) under stirring until the formation of a clear solution. In the above solution, 40 ml of 2 M Na_2_CO_3_ aqueous solution was then added dropwise under stirring at room temperature. After being aged for 6 h, the suspension liquid was filtrated, washed more than 50 times with DI water, and dried overnight at 90 °C. The resulting samples were calcinated in muffle furnace at 450 °C for 2 h.

### Synthesis of ZnFe_2_O_4_ oxide

ZnFe_2_O_4_ catalyst was synthesized by co-precipitation method. In total, 0.1 mol/l Fe(NO_3_)_3_·9H_2_O and 0.1 mol/l Zn(NO_3_)_2_·6H_2_O were thoroughly mixed and preheated to 80 °C. Thereafter, 1 mol/l Na_2_CO_3_ was added dropwise to the mixture and continuous stirred at the speed of 720 rpm, and it would be aging for 5 h at the pH around 10. The filter cake was obtained from suction filtration operation of aged solution, and washed more than 50 times to exclude the surfactant. Then the precipitate was dried out overnight in the oven at 80 °C. After cooling to the room temperature, the catalyst was sent to muffle furnace after sufficient grinding. The temperature was raised from room temperature to 450 °C in 120 min.

### Synthesis of LaFeO_3_ oxide

LaFeO_3_ samples were synthesized using the microemulsion technique. Initially, three distinct microemulsions were formulated, each with varying aqueous phases: the first contained Lanthanum (III) chloride heptahydrate (0.19 mmol LaCl_3_·7H_2_O), the second had iron (III) chloride tetrahydrate (0.20 mmol FeCl_3_·4H_2_O), and the third comprised 0.45 mmol NH_4_OH. Each microemulsion was stabilized with a consistent 0.12 M concentration of dioctyl sulfosuccinate sodium (aerosol-OT) and supplemented with isooctane as the oil phase. Subsequently, the Lanthanum-containing microemulsion was combined with the iron-enriched one to form an intermediate emulsion, designated as microemulsion I. After an hour of mechanical mixing, the NH_4_OH-containing microemulsion was introduced into microemulsion I. This mixture underwent vigorous stirring at ambient conditions for 2 h, facilitating the formation of a LaFeO_3_ precursor. Methanol was later added to induce phase separation and serve as a washing agent. After more than 50 times washing cycles, the solution was centrifuged to isolate the LaFeO_3_ precursor, which was dried overnight at 80 °C. The final crystalline LaFeO_3_ product was obtained post-calcination at 450 °C for 2 h.

### Synthesis of nano-sized-H-ZSM-5

Nano-sized H-ZSM-5 with different Si/Al ratio was prepared by using the hydrothermal method. Appropriate amounts of Tetraethyl orthosilicate (TEOS (11.2 g)), Tetra propylammonium hydroxide (TPAOH (13.1 g)), Aluminum nitrate (Al(NO_3_)_3_·9H_2_O (0.6/0.52/0.45/0.3/0.15 g)), Sodium hydroxide (NaOH (0.1 g)), Propan-2-ol (IPA (0.1 g)), and urea (2 g) were dissolved in DI water (18.4 g) under vigorous stirring for at least 6 h, at room temperature. The solution was transferred into a Teflon-lined stainless steel autoclave reactor that was further placed in a temperature-programmed oven. For crystallization, the temperature of the oven was raised from room temperature to 180 °C with a heating rate of 15 °C/h and held at 180 °C for 48 h. Once the crystallization is complete, the temperature of the autoclave reactor was quenched rapidly using a cold water bath. The obtained crystals were then collected through the filtration process (washed several times with DI water to remove the mother liquid) and dried overnight in the oven at 90 °C. To remove the templating agent TPAOH, the crystals were calcined in air at 550 °C for 5 h. Using a cation exchange method (step repeated at least three times), the Na-form of ZSM-5 (i.e., 10 g) was converted to NH4-ZSM-5 after being dispersed in a 1 M NH4NO3 solution (i.e., 100 ml) under vigorous stirring for 6 h. Finally, NH_4_-ZSM-5 was transformed into H-ZSM-5 through a simple calcination step performed at 550 °C for 5 h in the environment of air. The resulting powder was denoted as H-ZSM-5.

### Catalytic performance evaluation

CO_2_ hydrogenation reactions were carried out in a fixed-bed reactor under 30 bar of mixed gas. The mixed gas with a H_2_/CO_2_ ratio of 3:1 (gas composition, 3.18% Ar, 5.42% CO, 22.85 CO_2_, and 68.55% H_2_) unless otherwise stated. Generally, the catalyst with grain size of 355–850 um (1 g, 20–40 meshes) diluted with powdered quartz was loaded into a fixed-bed reactor with an inner diameter of 10 mm. The reaction was carried out at 350 °C, 3.0 MPa and 1000 ml g^−1^ h^−1^ unless otherwise specified. All the reaction products were kept in the gas phase and analyzed online by two gas chromate-graphs (Agilent 7890A) equipped with a HP-PLOT/Q capillary column connected to a flame ionization detector (FID) and a TDX-1 column (produced from DICP) connected to a thermal conductivity detector (TCD). CH_4_ was taken as a reference bridge between FID and TCD. Ar was used as an inner standard. The CO_2_ conversion and the CO selectivity, hydrocarbons (C_n_H_m_), MeOH and DME selectivity among the carbon products without CO (including C_n_H_m_, MeOH and DME) were calculated with the followed equations:

1 $${{{{{{\rm{CO}}}}}}}_{2} \, {{{{{\rm{conversion}}}}}}=({{{{{{\rm{CO}}}}}}}_{2{{{{{\rm{in}}}}}}}-{{{{{{\rm{CO}}}}}}}_{2{{{{{\rm{out}}}}}}})/({{{{{{\rm{CO}}}}}}}_{2{{{{{\rm{in}}}}}}})\times 100\%$$

CO_2in_: moles of CO_2_ at the inlet; CO_2out_: moles of CO_2_ at the outlet;

2 $${{{{{\rm{CO\; selectivity}}}}}}=\, {{{{{{\rm{CO}}}}}}}_{{{{{{\rm{out}}}}}}}/({{{{{{\rm{CO}}}}}}}_{2{{{{{\rm{out}}}}}}}-{{{{{{\rm{CO}}}}}}}_{2{{{{{\rm{out}}}}}}})\times 100\%$$

CO_out_: moles of CO at the outlet;

3 $${{{{{{\rm{C}}}}}}}_{{{{{{\rm{n}}}}}}}{{{{{{\rm{H}}}}}}}_{{{{{{\rm{m}}}}}}}{{{{{\rm{selectivity}}}}}}={{{{{\rm{n}}}}}}{{{{{{\rm{C}}}}}}}_{{{{{{\rm{n}}}}}}}{{{{{{\rm{H}}}}}}}_{{{{{{\rm{m\; outlet}}}}}}}/\Sigma {{{{{\rm{i}}}}}}{{{{{{\rm{C}}}}}}}_{{{{{{\rm{i}}}}}}}{{{{{{\rm{H}}}}}}}_{{{{{{\rm{m}}}}}}}{{{{{\rm{outlet}}}}}}\times 100\%$$

where C_n_H_m_ outlet represents moles of individual hydrocarbon product at the outlet. The carbon balance was between 95.0 and 102.0%.

### In situ diffuse reflectance Fourier transform infrared spectroscopy (DRIFTS) measurement

The change of reaction species was explored with an in situ DRIFTS method. The catalyst wafer was reduced in hydrogen (6% in volume, N_2_ balanced) at 20 ml/min and 350 °C for 1 h, and then swept in nitrogen at the same flow rate and temperature for 20 min. The DRIFTS spectra were recorded with a Nicolet 6700 spectrometer by collecting 64 scans at 4 cm^−1^ resolution. After that, the catalyst was reacted with (9 vol.% H_2_ + 3 vol.% CO_2_, N_2_ balanced) at 10 ml/min, 350 °C, and atmospheric pressure. The in situ DRIFTS spectra were recorded every 5 min until the reaction lasted for 60 min.

### Catalyst characterizations

The morphologies of samples were performed by field-emission scanning electron microscopy (FESEM, SU-8010), transmission electron microscopy (TEM, HT7700 120 kV).

The atomic scale scan transmission electron microscopy (STEM) high angle annular dark field (HAADF) experiments were performed using a Cs-corrected scanning transmission electron microscope (FEI Titan Cubed Themis G2 300) operated at 300 kV with a convergence semi-angle of 23.6 mrad and the beam current was lower than 30 pA. The microscope was equipped with a DCOR+ spherical aberration corrector for the electron probe which was aligned before the experiments using a standard gold sample. The dwell time of probe scanning was 32 μs for all the EDS mapping image. The dwell time was 1 µs per pixel with a map size of 256 × 256 pixels; a complete process of EDS mapping took roughly 0.5 h to reach an appropriately high signal-to-noise ratio. The following aberration coefficients were measured as: A1 = 694 pm; A2 = 22.1 nm; B2 = 6.12 nm; C3 = 82.5 nm; A3 = 63.6 nm; S3 = 136 nm; A4 = 2.13 μm, D4 = 4.54 μm, B4 = 1.71 μm, C5 = −49.2 μm, A5 = 222 μm, S5 = 102 μm and R5 = 17.3 μm.

Atomic-resolution integrated differential phase contrast (iDPC-STEM) was also operated at 300 kV with a convergence semi-angle was 15 mrad. The beam current was set between 1 pA and 0.5 pA. The iDPC-STEM experiments were performed using a Cs-corrected scanning transmission electron microscope (FEI Titan Cubed Themis G2 300) operated at 300 kV. The microscope was equipped with a DCOR+ spherical aberration corrector for the electron probe which was aligned before the experiments using a standard gold sample. The following aberration coefficients were measured as: A1 = 1.41 nm; A2 = 11.5 nm; B2 = 22.2 nm; C3 = 2.05 μm; A3 = 525 nm; S3 = 177 nm; A4 = 8.81 μm, D4 = 2.39 μm, B4 = 13.2 μm, C5 = −3.95 mm, A5 = 295 μm, S5 = 111 μm and R5 = 102 μm. The convergence semi-angle was 15 mrad, the beam current was lower than 0.5 pA (the measurement was limited by the precision of the Faraday cup), the collection angle was 4–22 mrad, and the dwell time of probe scanning was 32 μs.

The X-ray diffraction patterns of various metal oxides and zeolites were obtained by Bruker D8 Advance X-ray diffractometer using CuKα radiation (*λ* = 1.5418 Å) at 40 mA and 40 kV. X-ray photoelectron spectroscopy (XPS) of samples was tested on Escalab 250 Xi XPS with an Al Kα X-ray resource (Thermo Fisher Scientific). The Brunauer-Emmett-Teller surface area of samples were performed by specific surface area and mesoporous analyzer (TriStar II).

The X-ray absorption fine structures (XAFS) measurements were analyzed at the BL14W1 station in Shanghai Synchrotron Radiation Facility (SSRF, 3.5 GeV, 250 mA in maximum, Si (111) double-crystals). The energy was calibrated accordingly to the absorption edge of pure Fe foil. Before XAFS experiments, the as-prepared samples were transferred to a glove box for tableting without exposure to air. All XANES and EXAFS data were processed using Athena program.

Aldol-Aromatics composition retained inside the spent catalyst was determined by GC-MS using the hydrofluoric dissolution technique. Appropriate amounts of hydrofluoric acid (10–20%) was slowly and carefully added to 1 g of physically mixed composite catalyst (LaFeO_3_ and H-ZSM-5). Then a few drops of methylene chloride (CH_2_Cl_2_) were added to the above solution and centrifuged until a layer of separated hydrocarbon oil was visible. This oil was then carefully extracted from the mother solution and analyzed on a GC-MS. These steps were repeated on samples obtained at different times on stream.

### Theoretical methods

Density functional theory (DFT) calculations were performed by using the revised Perdew–Burke–Ernzerhof (RPBE) functional within the generalized gradient approximation (GGA) implemented in Vienna Ab Initio Simulation Package^57,58^. The projector-augmented wave method was applied to describe the electron–ion interactions^54^, and the D3 Grimme’s method was employed to correct van der Waals interaction. We used a plane-wave cutoff energy of 400 eV and the Gaussian smearing with a width of 0.1 eV. Periodic boundary conditions were applied, and more than 20 Å of vacuum space was used to avoid the interaction of the adjacent images.

The adsorption energies were evaluated using eight-layer 2 × 2 supercells with the bottom four layers constrained, and [4 × 4 × 1] Monkhorst-Pack k-point grids were used with a convergence threshold of 10^–6^ eV for the iteration in self-consistent field (SCF)^59^. All structures were optimized until force components were less than 0.01 eV/Å. The vibrational frequencies of free molecules and adsorbates were calculated by using the phonon modules in the VASP 5.4.4 code. A standard thermodynamic correction was applied to determine the free energy corrections, including the correction of the effect from zero-point energy, pressure, inner energy, and entropy. All the formation energies of surface adsorbates/states, as well as gas-phase products were referenced to CO_2_(g), H_2_(g), and H_2_O(g). Due to the DFT errors in estimating gas-phase molecules containing O=C=O or O–C=O backbone, such as CO_2_(g) and HCOOH(g), at a PBE/RPBE level, a double bond correction of +0.33 eV was applied to raw energies of CO_2_(g) and HCOOH(g). The way of obtaining such a correction is based on previous studies^60,61^. We calculated the transition state using the climbing image nudged elastic band (CI-NEB) method. The vibrational frequencies of the transition state are also calculated and it should be noted that there is only one imaginary frequency.

We used adsorption free energies, Δ*G*, instead of electronic energies to construct Gibbs free energy diagram. Here we made the assumption that the gaseous products in the pathway were calculated at partial pressures as Fig. 2a depicted. The enthalpy, entropy and Gibbs free energy of each species were calculated by vibrational frequency analysis based on harmonic normal mode approximation using the finite difference method in VASP 5.4.4. The Gibbs free energy for a given species is *G*(*T*, *P*) = *E*_e_ + *E*_trans_ + *E*_rot_ + *E*_vib_ + PV-T(*S*_trans_ + *S*_rot_ + *S*_vib_) Where

$${{{E}}}_{{{{{{\rm{trans}}}}}}}=3/2{{{{{\rm{RT}}}}}}$$

$${{{E}}}_{{{{{{\rm{rot}}}}}}}={{{{{\rm{RT}}}}}}({{{{{\rm{for}}}}}}\; {{{{{\rm{linear}}}}}}\; {{{{{\rm{molecule}}}}}})$$

$${{{E}}}_{{{{{{\rm{vib}}}}}}}=R\sum \frac{{hvn}}{{kb}} \left(\frac{1}{2}+\frac{1}{{e}^{{hvn}/{kbT}}-1}\right)$$

$${{{S}}}_{{{{{{\rm{trans}}}}}}}={{R}}\left({{{{{\rm{ln}}}}}}{q}_{{{{{\rm{trans}}}}}}+\frac{5}{2}\right)$$, where $${{{q}}}_{{{{{{\rm{trans}}}}}}}={(2\Pi {{mkbT}}/{{{h}}}^{2})}^{3/2}{{{k}}}_{{{b}}}{{T}}/{{P}}$$

$${{{S}}}_{{{{{{\rm{rot}}}}}}}={{R}}({{{{{{\rm{In}}}}}}}{q}_{{{{{{\rm{rot}}}}}}}+1)\, ({{{{{\rm{for}}}}}}\; {{{{{\rm{linear}}}}}}\; {{{{{\rm{molecule}}}}}})$$

where $${{{q}}}_{{{{{{\rm{rot}}}}}}}=1/{{\sigma }}\, (8{\Pi }^{2}{{kbT}}/{{{h}}}^{2})\times {{I}}$$

$${{{S}}}_{{{{{{\rm{rot}}}}}}}={{R}}({{{{{{\rm{In}}}}}}}{q}_{{{{{{\rm{rot}}}}}}}+3/2) \, ({{{{{\rm{for}}}}}}\; {{{{{\rm{nonlinear}}}}}}\; {{{{{\rm{molecule}}}}}}),$$

where $${q_{rot}}=\frac{\sqrt{\prod }}{\sigma }\, {\left(\frac{8{\prod }^{2}{kbT}}{h2}\right)}^{3/2}\times \sqrt{{Ix}*{Iy}*{Iz}}$$

$${{S}}_{{{{{{{\rm{vib}}}}}}}}=R\sum \frac{{hvn}/{kbT}}{{e}^{{hvn}/{kbT}}-1}-{{{{\mathrm{ln}}}}}(1-{e}^{-{hvn}/{kbT}})$$

where *I* is the moment of inertia, *σ* is the rotational symmetry number, and *m* is the mass of the molecule. For adsorbed molecules and transition states on the surface, the rotational and translational contributions were converted into vibration modes. We also approximated that the PV term of the surface species is negligible because it is very small with regard to the energetic terms, and thus, we considered *G*(*T*,*P*) = *E*_e_ + *E*_vib_ − *T* × *S*_vib_ in this case.

The stability of Fe_2_O_3_ and LaFeO_3_ with respect to Fe_5_C_2_ were determined with the following equations:

$$5\,{{{{{\rm{LaFe}}}}}}{{{{{{\rm{O}}}}}}}_{3}+2\,{{{{{{\rm{CO}}}}}}}_{2}+23/2\,{{{{{{\rm{H}}}}}}}_{2}\to 1\,{{{{{{\rm{Fe}}}}}}}_{5}{{{{{{\rm{C}}}}}}}_{2}+5/2\,{{{{{{\rm{La}}}}}}}_{2}{{{{{{\rm{O}}}}}}}_{3}+23/2 \, {{{{{{\rm{H}}}}}}}_{2}{{{{{\rm{O}}}}}}$$

$$5 \, {{{{{\rm{LaFe}}}}}}{{{{{{\rm{O}}}}}}}_{3}+2 \, {{{{{\rm{CO}}}}}}+19/2 \, {{{{{{\rm{H}}}}}}}_{2}\to 1 \, {{{{{{\rm{Fe}}}}}}}_{5}{{{{{{\rm{C}}}}}}}_{2}+5/2 \, {{{{{{\rm{La}}}}}}}_{2}{{{{{{\rm{O}}}}}}}_{3}+19/2 \, {{{{{{\rm{H}}}}}}}_{2}{{{{{\rm{O}}}}}}$$

$$5/2\, {{{{{{\rm{Fe}}}}}}}_{2}{{{{{{\rm{O}}}}}}}_{3}+2 \, {{{{{{\rm{CO}}}}}}}_{2}+23/2 \, {{{{{{\rm{H}}}}}}}_{2}\to 1 \, {{{{{{\rm{Fe}}}}}}}_{5}{{{{{{\rm{C}}}}}}}_{2}+23/2 \, {{{{{{\rm{H}}}}}}}_{2}{{{{{\rm{O}}}}}}$$

$$5/2 \, {{{{{{\rm{Fe}}}}}}}_{2}{{{{{{\rm{O}}}}}}}_{3}+2 \, {{{{{\rm{CO}}}}}}+19/2 \, {{{{{{\rm{H}}}}}}}_{2}\to 1 \, {{{{{{\rm{Fe}}}}}}}_{5}{{{{{{\rm{C}}}}}}}_{2}+19/2 \, {{{{{{\rm{H}}}}}}}_{2}{{{{{\rm{O}}}}}}$$

$$5/2 \, {{{{{\rm{Zn}}}}}}{{{{{{\rm{Fe}}}}}}}_{2}{{{{{{\rm{O}}}}}}}_{4}+2 \, {{{{{\rm{CO}}}}}}+19/2 \, {{{{{{\rm{H}}}}}}}_{2}\to 5/2 \, {{{{{\rm{ZnO}}}}}}+{{{{{{\rm{Fe}}}}}}}_{5}{{{{{{\rm{C}}}}}}}_{2}+19/2 \, {{{{{{\rm{H}}}}}}}_{2}{{{{{\rm{O}}}}}}$$

$$5/2 \, {{{{{\rm{Zn}}}}}}{{{{{{\rm{Fe}}}}}}}_{2}{{{{{{\rm{O}}}}}}}_{4}+4 \, {{{{{{\rm{CO}}}}}}}_{2}+23/2 \, {{{{{{\rm{H}}}}}}}_{2}\to 5/2 \, {{{{{\rm{ZnO}}}}}}+{{{{{{\rm{Fe}}}}}}}_{5}{{{{{{\rm{C}}}}}}}_{2}+23/2 \, {{{{{{\rm{H}}}}}}}_{2}{{{{{\rm{O}}}}}}$$

The Gibbs free energy change of the above reactions were adopted to quantify the likelihood of Fe_2_O_3_, ZnFe_2_O_4_ and LaFeO_3_ to form Fe_5_C_2_.

### Supplementary information

Supplementary Information

Peer Review File

## Data Availability

All data are available in the manuscript or the Supplementary Information. Additional data are available from the corresponding authors upon request.
