# Supplementary material for: Upgrading CO2 to sustainable aromatics via perovskite-mediated tandem catalysis
Source: Nat Commun. 2024 Apr 8;15:3037. doi: 10.1038/s41467-024-47270-z (PMC11002022; doi:10.1038/s41467-024-47270-z)
Supplement: Supplementary file 1 — Supplementary Information [file 41467_2024_47270_MOESM1_ESM.docx]

**Upgrading CO_2_ to sustainable aromatics via perovskite-mediated tandem catalysis**

**Authors:** *Guo Tian^1^*^†^, *Zhengwen Li^1^*^†^*, Chenxi Zhang^1,2,3*^, Xinyan Liu^4^, Xiaoyu Fan^1^, Kui Shen^5^, Haibin Meng^6^, Ning Wang^7^, Hao Xiong^1^, Mingyu Zhao^1^, Xiaoyu Liang^1^, Liqiang Luo^1^*, *Lan Zhang^7^, Binhang Yan^1^, Xiao Chen^1,2*^, Hong-Jie Peng^4*^, Fei Wei^1,2*^*

**Affiliations:**

1. Beijing Key Laboratory of Green Chemical Reaction Engineering and Technology, Department of Chemical Engineering, Tsinghua University, Beijing, China, 100084
2. Ordos Laboratory, Ordos, Inner Mongolia 017010, China
3. Institute for Carbon Neutrality, Tsinghua University, Beijing, 100084 China
4. Institute of Fundamental and Frontier Sciences, University of Electronic Science and Technology of China, Chengdu 611731, Sichuan, China
5. Key Laboratory of Fuel Cell Technology of Guangdong Province, School of Chemistry and Chemical Engineering, South China University of Technology, Guangzhou 510640, China
6. College of Chemistry, Taiyuan University of Technology, 030024 Taiyuan, China
7. Faculty of Environment and Life, Beijing University of Technology, Beijing 100124, China

**^†^** These authors contributed equally to this work

* Corresponding authors Chenxi Zhang, email: [cxzhang@tsinghua.edu.cn](mailto:cxzhang@tsinghua.edu.cn), Xiao Chen, email: [chenx123@tsinghua.edu.cn](mailto:chenx123@tsinghua.edu.cn), Hong-Jie Peng, email: [hjpeng@uestc.edu.cn](mailto:hjpeng@uestc.edu.cn), Fei Wei, email: [wf-dce@tsinghua.edu.cn](mailto:wf-dce@tsinghua.edu.cn).

**1. Supplementary Figures and Notes**

**
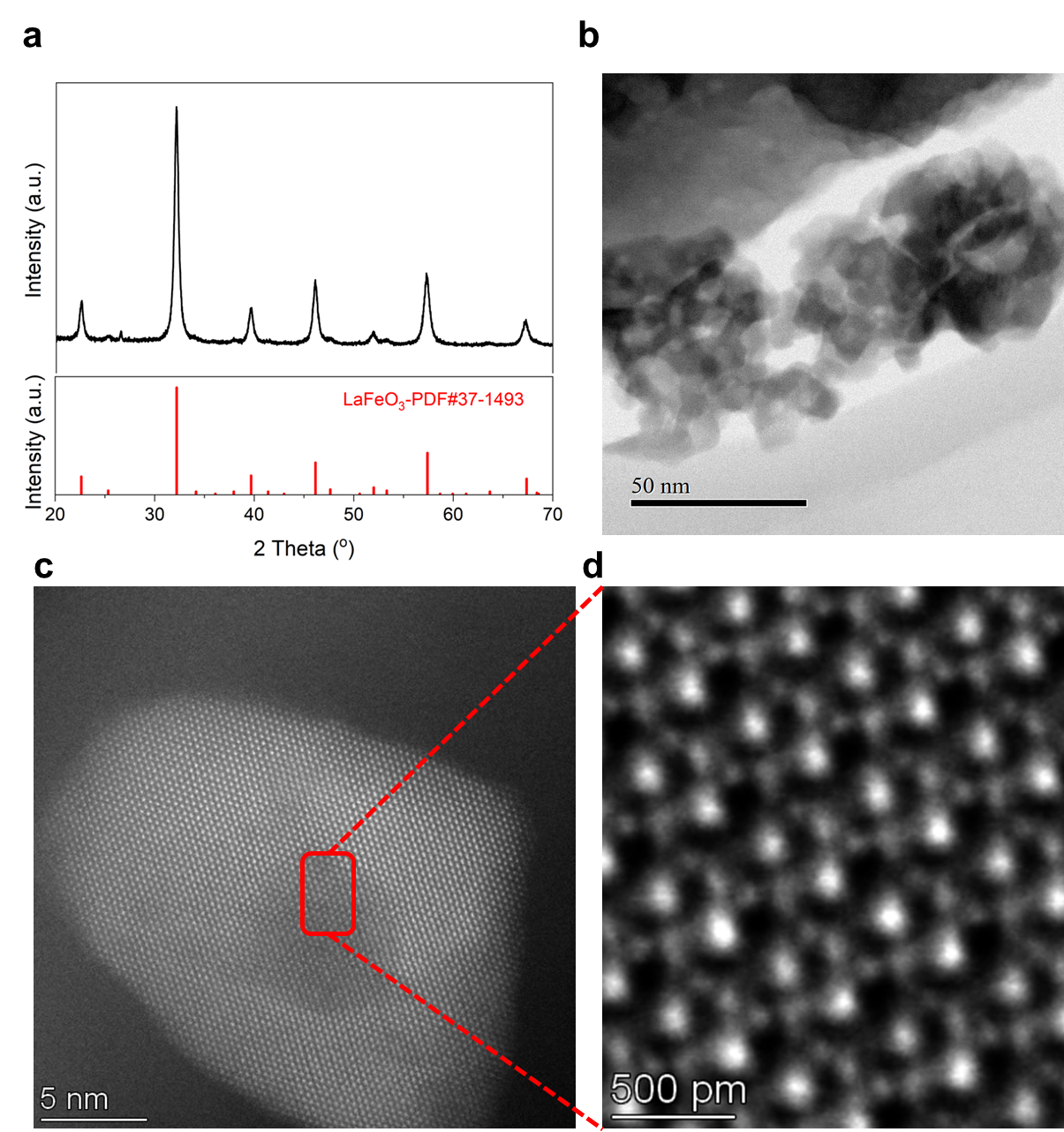
**

**Supplementary Fig. 1. Characterization of fresh LaFeO_3_.** (a) XRD. (b) TEM, (c) HAADF STEM, and (d) iDPC STEM images.

**
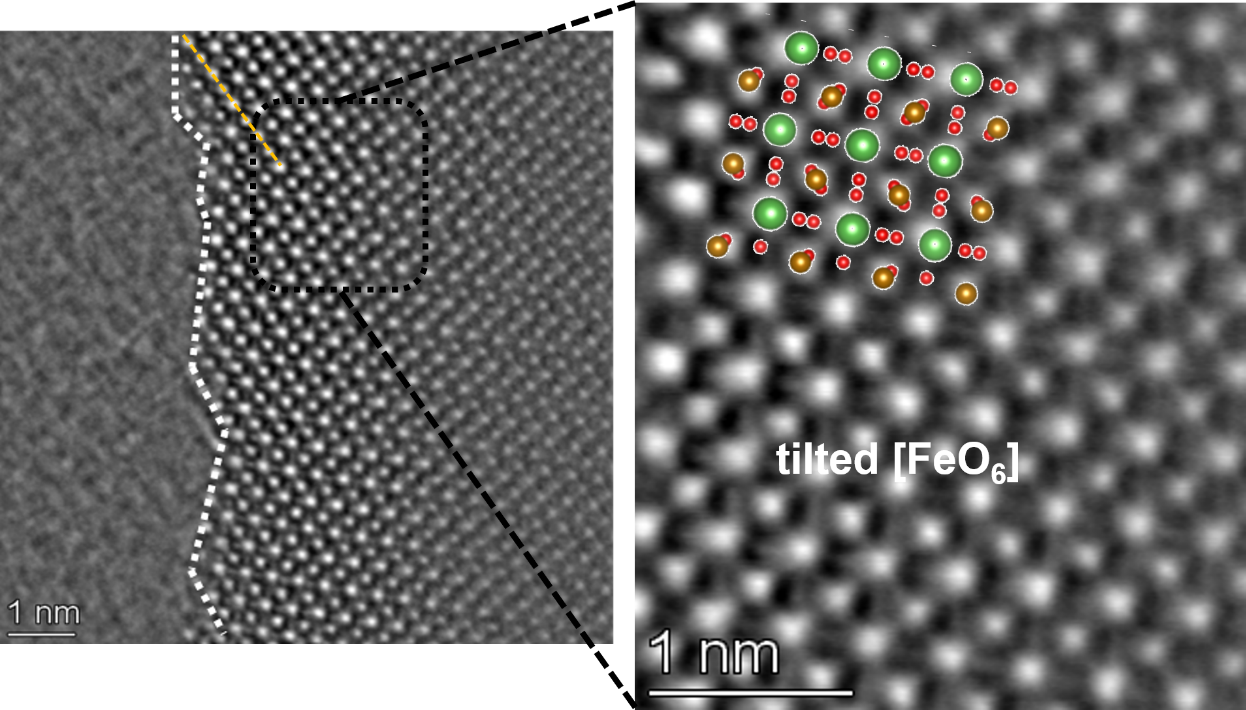
**

**Supplementary Fig. 2. Characterization of fine perovskite structure.** iDPC-STEM images of LaFeO_3_. The yellow line is used to guide eyes, indicating the deviation of top and bottom Fe–O bonds in a [FeO_6_] octahedron from the normal direction of the LaFeO_3_ (220) facet (the crystal cross-section as shown in the left image). Such deviation corresponds well with the tilting of [FeO_6_] octahedral, as indicated by the atomic structure on top of the magnification of selected region (right). Spheres in red, brown, and green represent O, Fe, and La atoms, respectively.


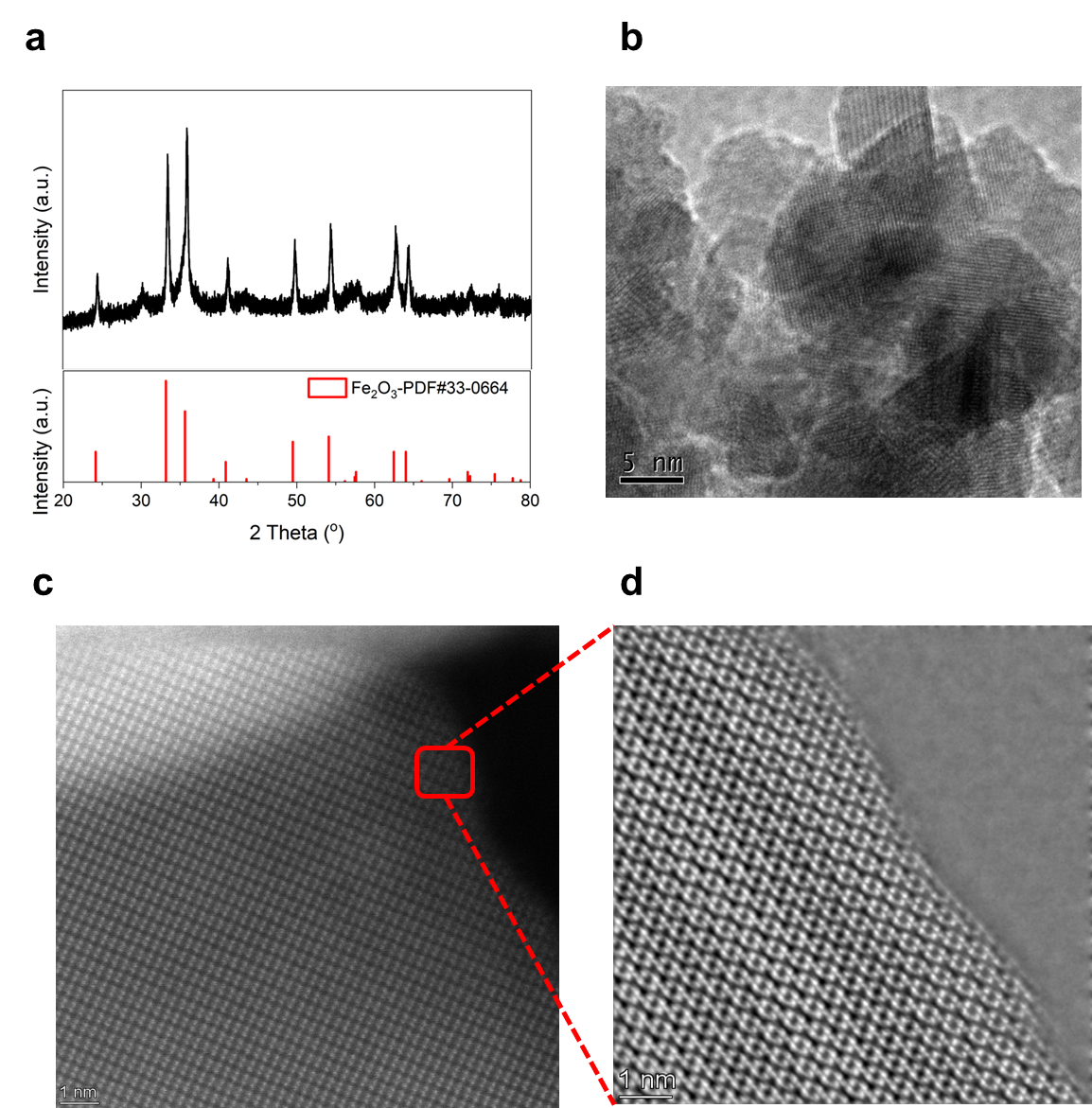


**Supplementary Fig. 3. Characterization of fresh Fe_2_O_3_.** (a) XRD. (b) TEM, (c) HAADF STEM, and (d) iDPC STEM images.


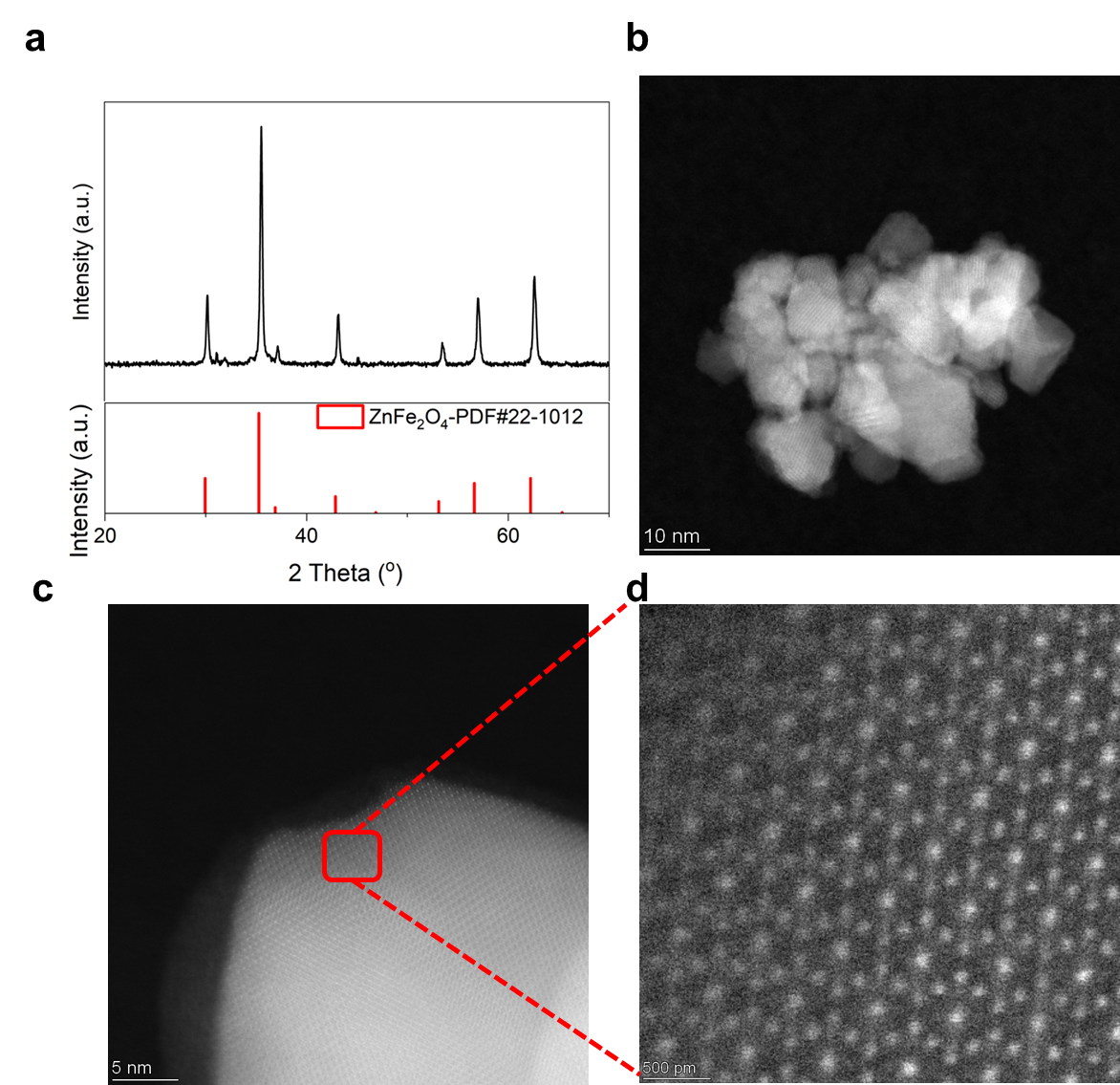


**Supplementary Fig. 4.** **Characterization of fresh ZnFe_2_O_4_.** (a) XRD. (b) TEM, (c) HAADF STEM, and (d) iDPC STEM images.


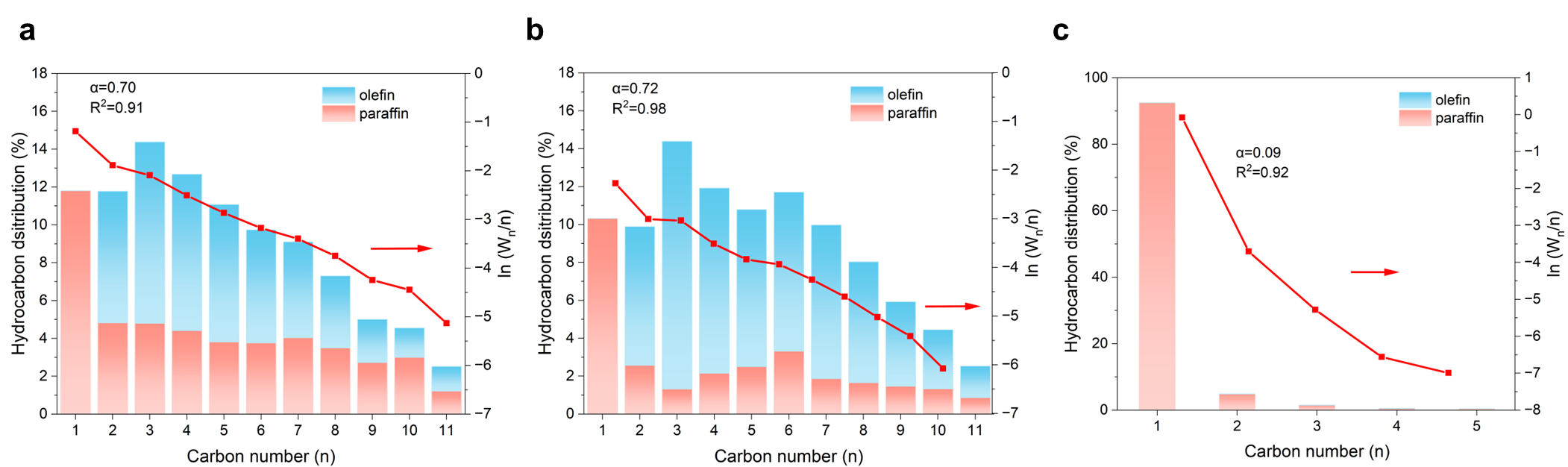


**Supplementary Fig. 5.** **Product distribution over three catalysts.** The detailed hydrocarbon product distribution, the ASF plot and the corresponding α value of (a) Fe_2_O_3_, (b) ZnFe_2_O_4_, and (c) LaFeO_3_.

**Note:** α is the probability of chain growth. R^2^ is the coefficient of determination, describing the goodness of liner fitting. W_n_ is the weight fraction of a product with n carbon atoms.


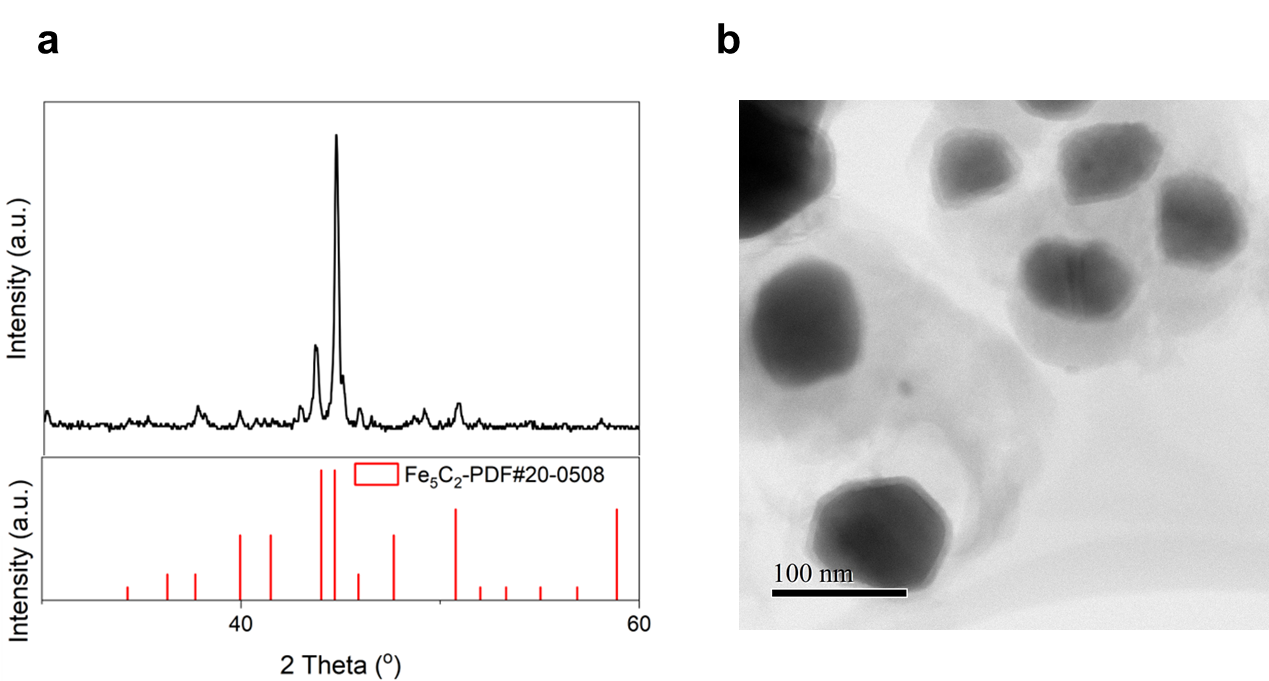


**Supplementary Fig. 6. Characterization of spent Fe_2_O_3_.** (a) XRD. (b) TEM image.

**Note:** The Fe_2_O_3_ was carburized to form the Fe_5_C_2_ phase after the reaction period of 24 h. Typically, the resultant Fe_5_C_2_ particles have a core–shell structure, in which polycrystalline Fe oxides and Fe_5_C_2_ constitute the shell and the core, respectively. The core–shell Fe_5_C_2_@Fe oxide particle could be as large as 100 nm, in contrast to 10–30 nm of original Fe_2_O_3_, corresponding to the catalyst sintering. The cocurrent carburization thus well explain the broad product distribution of pristine Fe_2_O_3_.


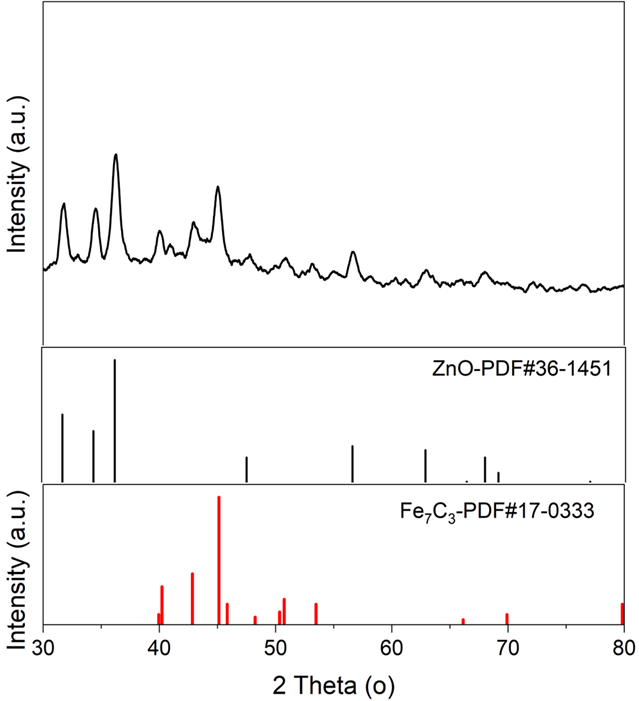


**Supplementary Fig. 7. Characterization of spent ZnFe_2_O_4_.** XRD spectra.

**Note:** After continuous reaction for 24 h, the initial ZnFe_2_O_4_ spinel crystals were completely transformed into ZnO and Fe_7_C_3_. According to research of conventional Fischer–Tropsch synthesis, Fe_7_C_3_ is also recognized as a catalyst for C–C coupling^11^.

**
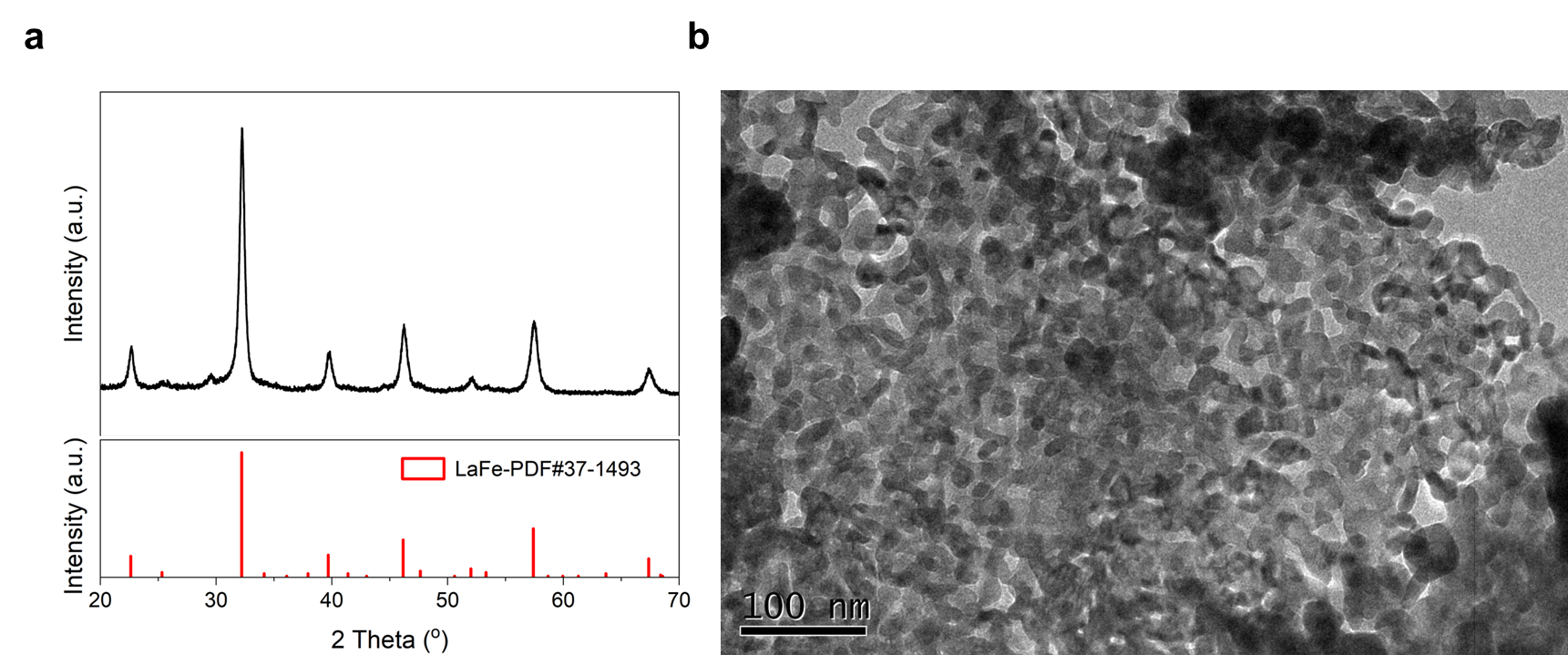
**

**Supplementary Fig. 8. Characterization of spent LaFeO_3_.** (a) XRD. (b) TEM image.


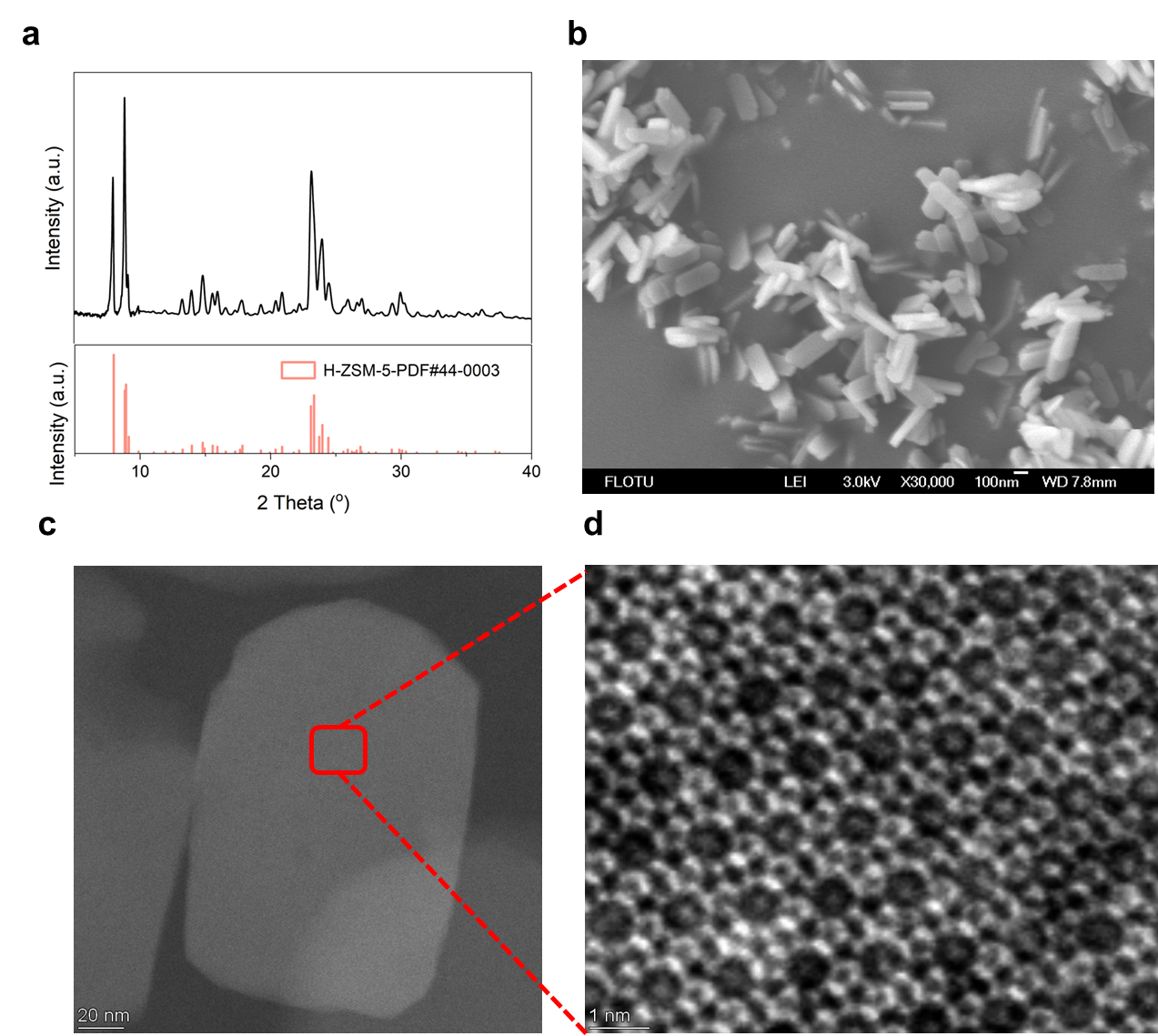


**Supplementary Fig. 9.** **Characterization of H-ZSM-5.** (a) XRD. (b) SEM, (c) HAADF STEM, and (d) iDPC-STEM images. **d** shows the [010] projection.


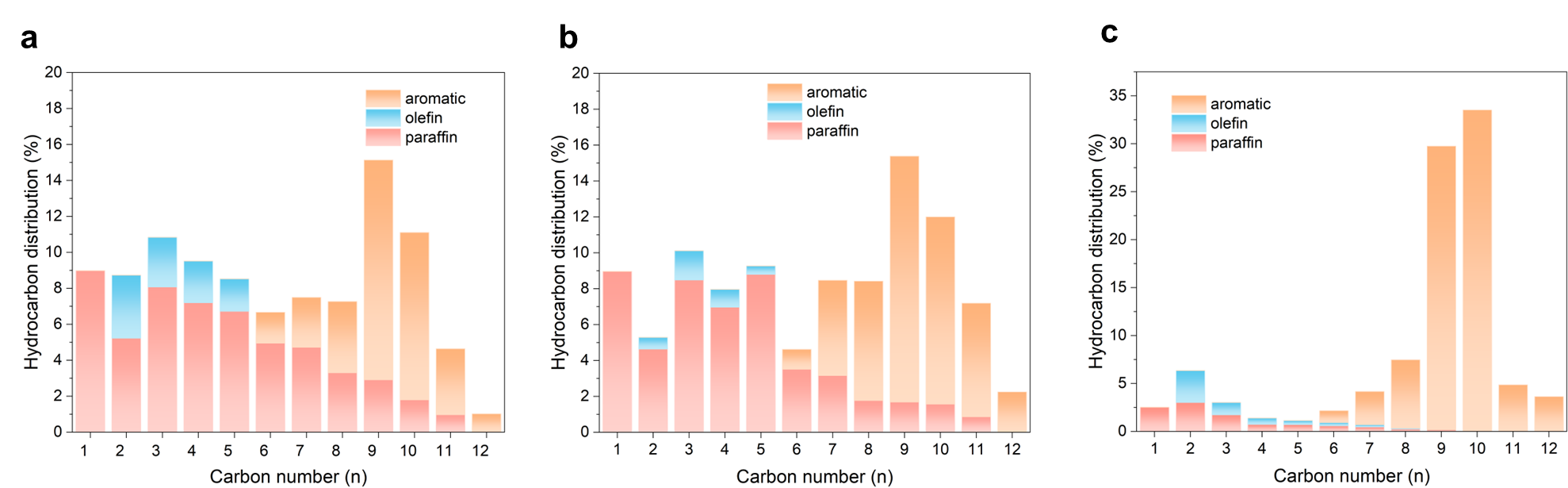


**Supplementary Fig. 10. Product distribution over composite catalyst.** The detailed hydrocarbon product distribution of (a) Fe_2_O_3_/H-ZSM-5, (b) ZnFe_2_O_4_/H-ZSM-5, and (c) LaFeO_3_/H-ZSM-5.

**
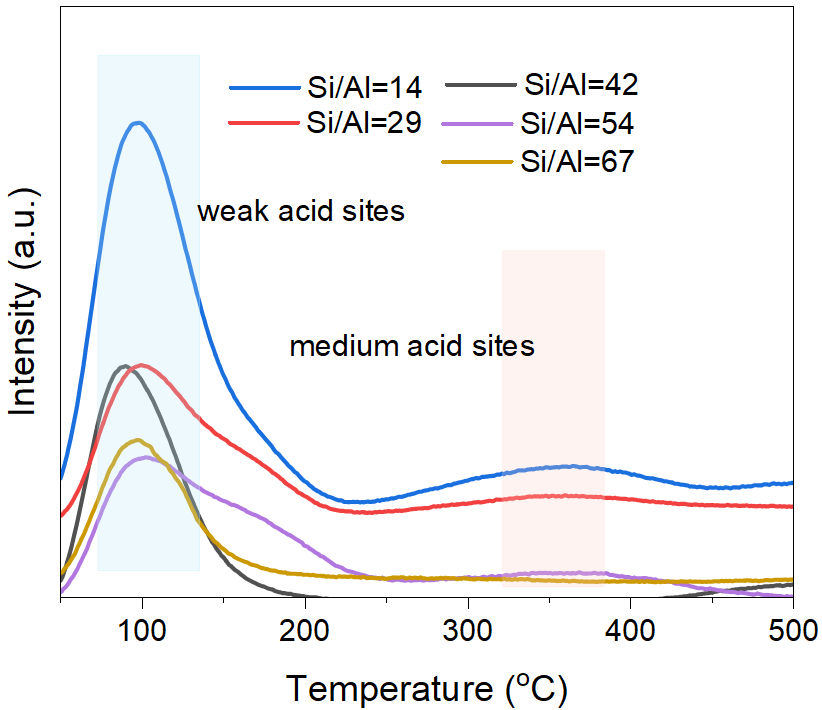
**

**Supplementary Fig. 11. NH_3_-TPD profiles for different Si/Al ratio of H-ZSM-5.** Deconvolution of the profiles indicates that the peak maximum being located below 200^o^C is attributed to NH_3_ desorption from weak acid sites while that in the range from 229-413^o^C to the medium strength acid sites. The NH_3_ desorption temperature reflects the acidity strength.


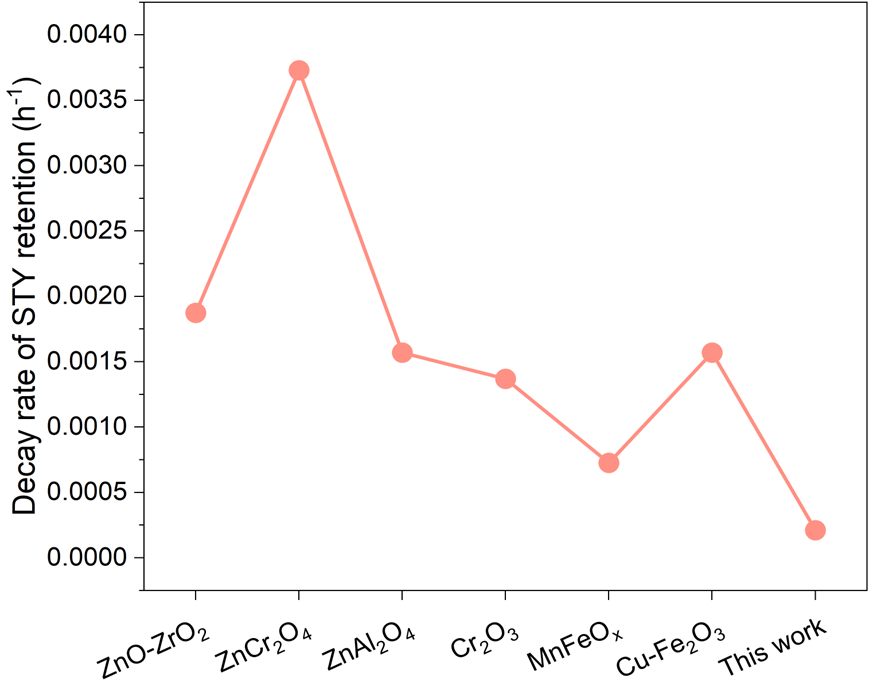


**Supplementary Fig. 12. Comparison between different catalysts.** In terms of the decay rate of STY (space time yield) per hour. The reported catalysts could be indexed in **Supplementary Table 1**.

**
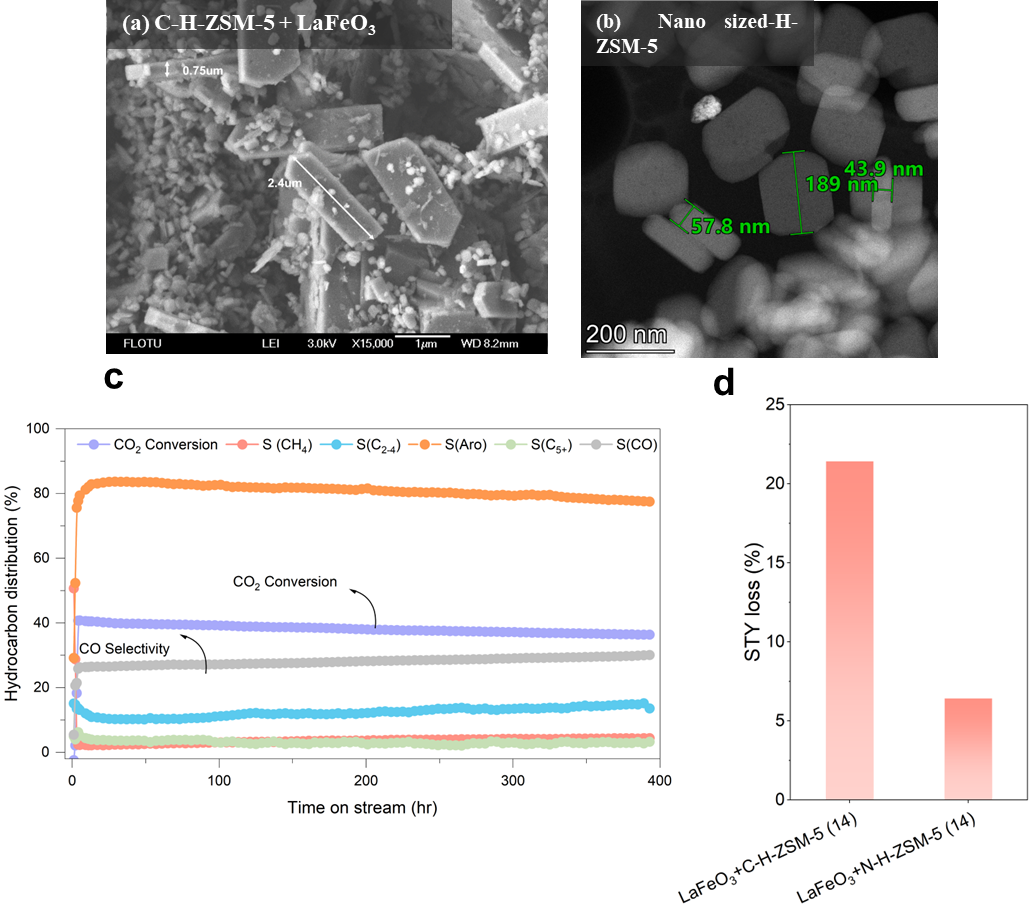
**

**Supplementary Fig. 13. Deactivation comparison between LaFeO_3_+Coventional H-ZSM-5 and LaFeO_3_+Nanosized H-ZSM-5.** (**a**) SEM images of C-H-ZSM-5 (**b**) HAADF-STEM image shows of nanosized H-ZSM-5. (**c**) Catalytic stability of LaFeO_3_+C-H-ZSM-5 over 6000ml/h/g_cat_, 350^o^C and 3.0Mpa, CO_2_/H_2_=1:6. (**d**) STY loss between LaFeO3+C-H-ZSM-5 with LaFeO_3_+N-H-ZSM-5.

**Note:** The conventional H-ZSM-5 exhibited large size with b-axis length > 600nm while the nanosized H-ZSM-5 exhibited a b-axis wit a length ranged from 50-75 nm. Such short b-axis length enables the fast diffusion of aromatic products, thus alleviating the deactivation rate of composite catalysts. As shown in **Supplementary Fig. 13**, after 400h, the composite catalysts with conventional H-ZSM-5 have decreased > 20% STY rate while the nanosized H-ZSM-5 with a decay rate ~6%. Such results demonstrate that the superior stability of our new reported perovskite-mediated OX-ZEO catalysts.


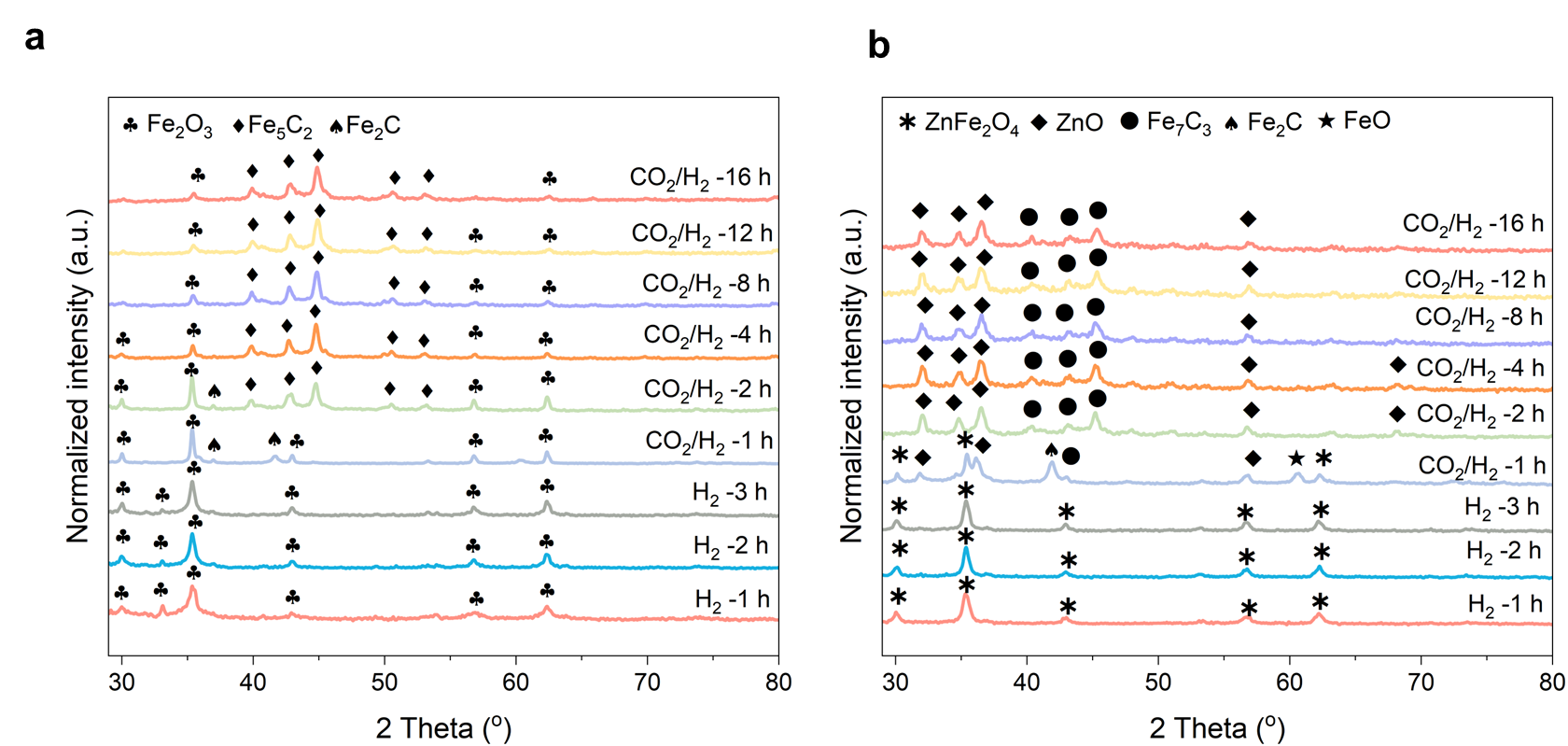


**Supplementary Fig. 14. In-situ XRD patterns.** (a) Fe_2_O_3_ and (b) ZnFe_2_O_4_ under H_2_ and CO_2_/H_2_ atmosphere at 350^o^C.

**Note:** Both oxides are shown with no new crystalline phases upon initial 3 h of H_2_ reduction. When exposing to carbonaceous reactive atmosphere (CO_2_/H_2_), Fe_2_O_3_ firstly transformed into a transition Fe_2_C phase and after reaction 16 h, Fe_2_O_3_ was completely carburized into Fe_5_C_2_. As for ZnFe_2_O_4_ spinel, when exposing to CO_2_/H_2_, ZnFe_2_O_4_ first decomposed and partially reduced to ZnO and FeO, then FeO was gradually carburized into Fe_2_C and finally Fe_7_C_3_. The standard XRD patterns of Fe_2_O_3_, Fe_2_C, Fe_5_C_2_, ZnFe_2_O_4_, ZnO, Fe_7_C_3_ were according to PDF # 33-0664, PDF # 17-0897, PDF # 20-0508, PDF # 22-1012, PDF # 36-1451, PDF # 17-0333.


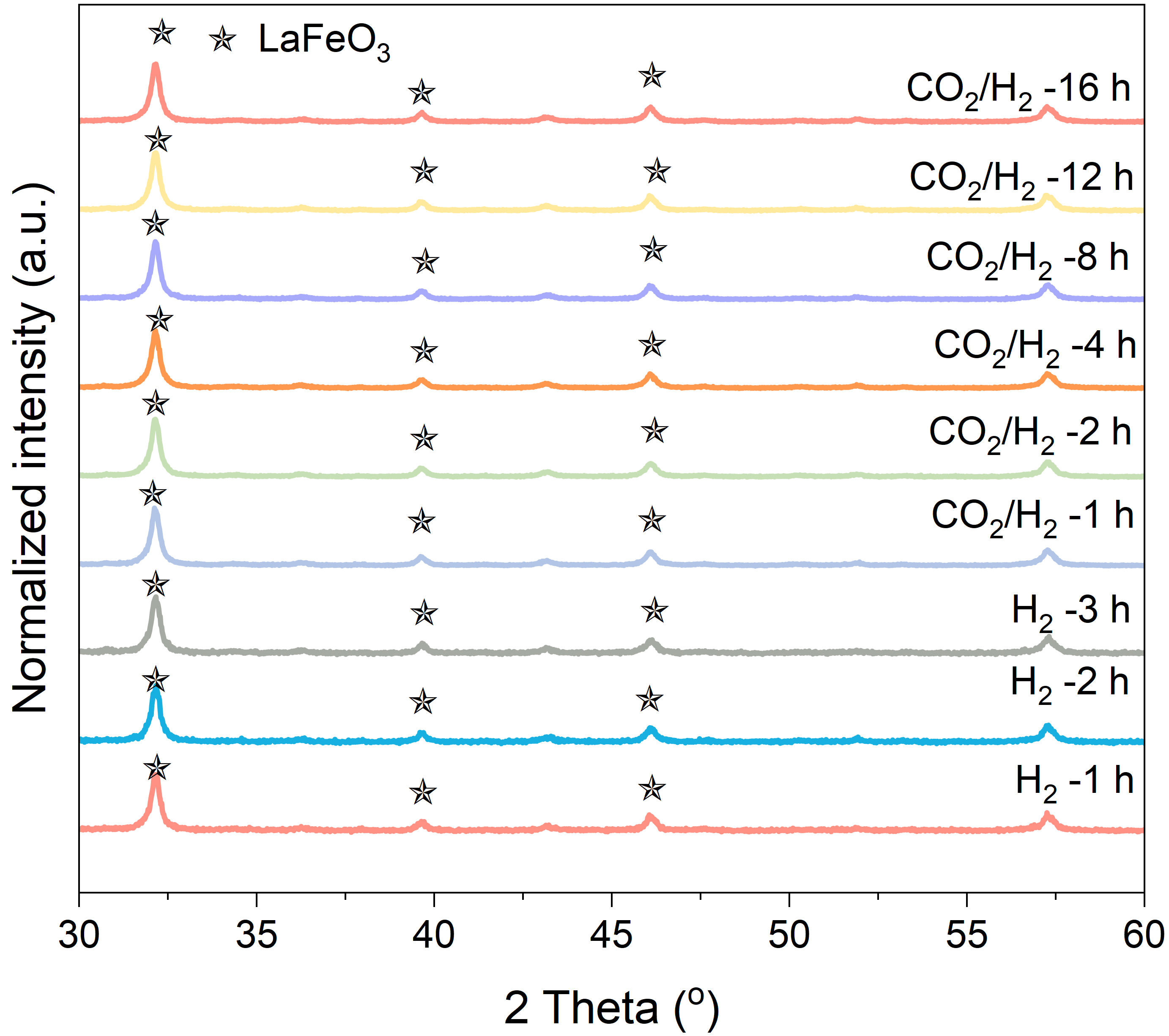


**Supplementary Fig. 15. In-situ XRD patterns of LaFeO_3_ under H_2_ rand CO_2_/H_2_ atmosphere at 350^o^C**. The standard XRD patterns were according to PDF# 37-1493.


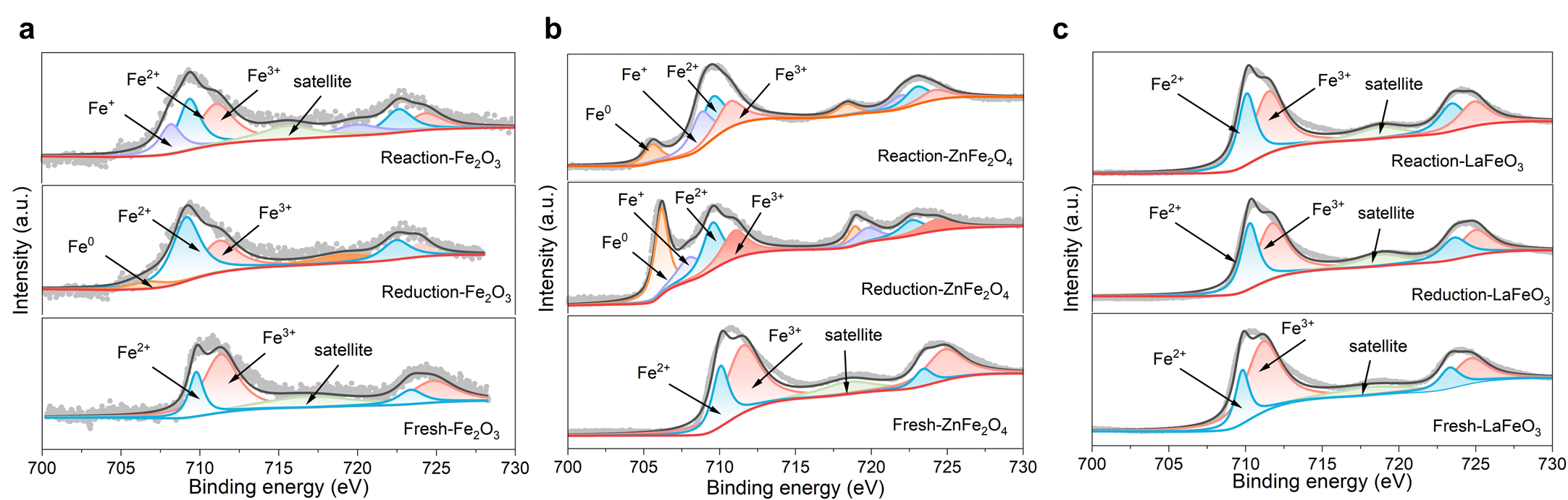


**Supplementary Fig. 16. In-situ Fe *2p* XPS spectra.** (a) Fe_2_O_3_, (b) ZnFe_2_O_4_, and (c) LaFeO_3_ catalyst denoted as fresh (bottom, before any treatment) after reduction (middle, after 4h H_2_ reduction), and reaction (top, further after 24h reaction under CO_2_/H_2_ atmosphere). The temperature for reduction and reaction was kept as 350^o^C.

**Note:** Although XRD reveals the bulk information of different iron oxides, in-situ X-ray photoelectron spectroscopy (XPS) technology were carried to probe surface Fe species under different atmosphere. As shown in **Supplementary Fig. 16a**, Fe^3+^ and unsaturated Fe^2+^ co-existed on the fresh Fe_2_O_3_. After H_2_ treatment for 4 h, amounts of Fe^2+^ and Fe^0^ appeared at the surface. The emergence of Fe^+^, which corresponds to the nominal valence of Fe in carbides, was observed after exposing to CO_2_/H_2_ for further 21 h. This is accompanied with disappearance of Fe^0^. A similar trend of the evolution of surface Fe species was also recognized for the ZnFe_2_O_4_ (**Supplementary Fig. 16b**). The slight difference is that Fe^0^ was only completely re-oxidized to Fe^+^. In sharp contrast to Fe_2_O_3_ and ZnFe_2_O_4_, no obvious Fe^+/0^ signal was detected during different atmospheres for LaFeO_3_ (**Supplementary Fig. 16c**). The increased amount of unsaturated Fe^2+^ is attributed to the increased number of oxygen vacancy (O_v_) upon reduction.


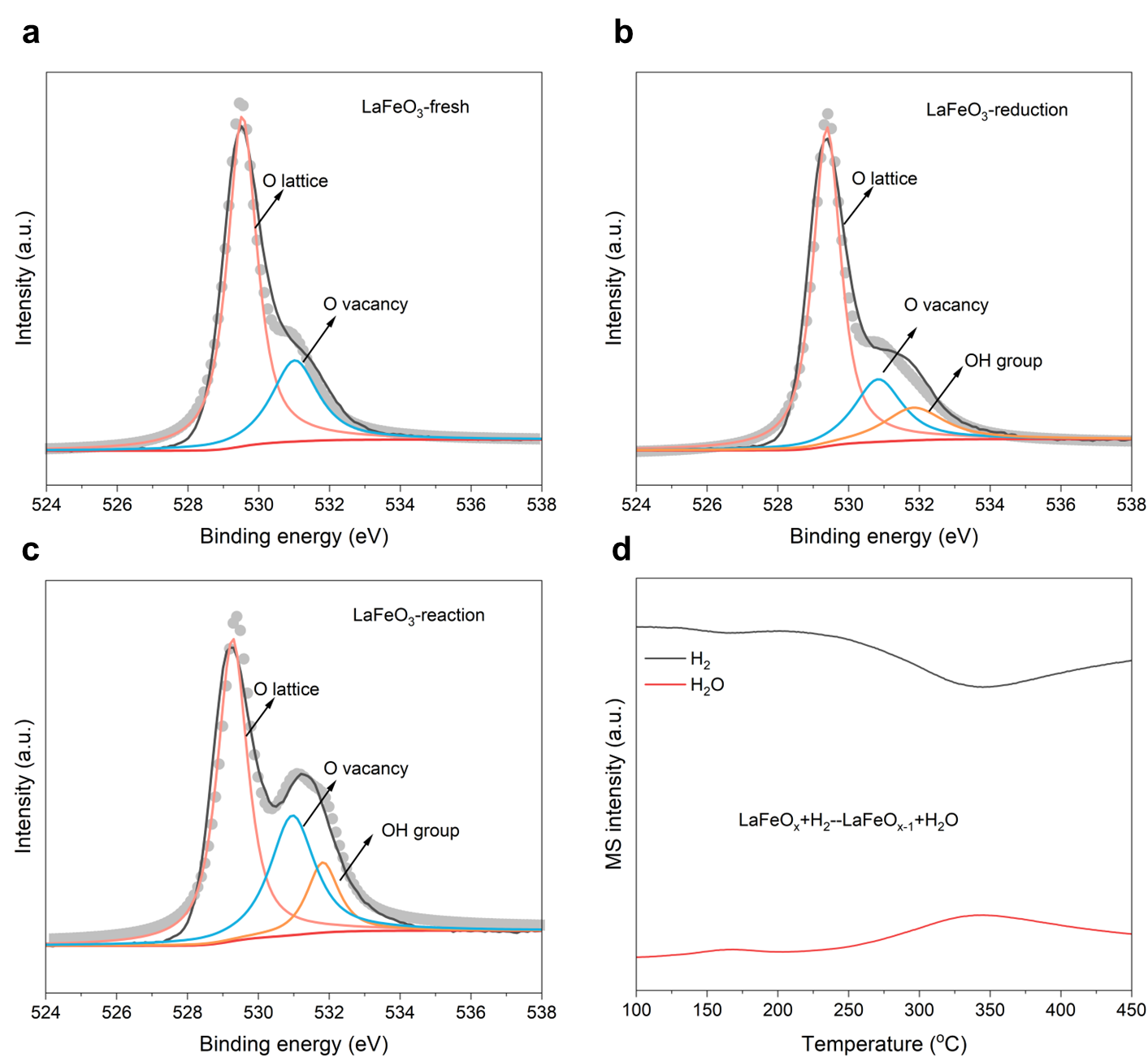


**Supplementary Fig. 17. In-situ O *1s* XPS spectra.** (a) fresh, (b) reduction, and (c) reaction states of LaFeO_3_. Nomenclature is the same as in **Supplementary Fig. 16**. (d) H_2_ temperature-programmed reduction (H_2_-TPR) experiment for LaFeO_3_ to validate the O_v_ formation (MS denote as the mass spectrum).


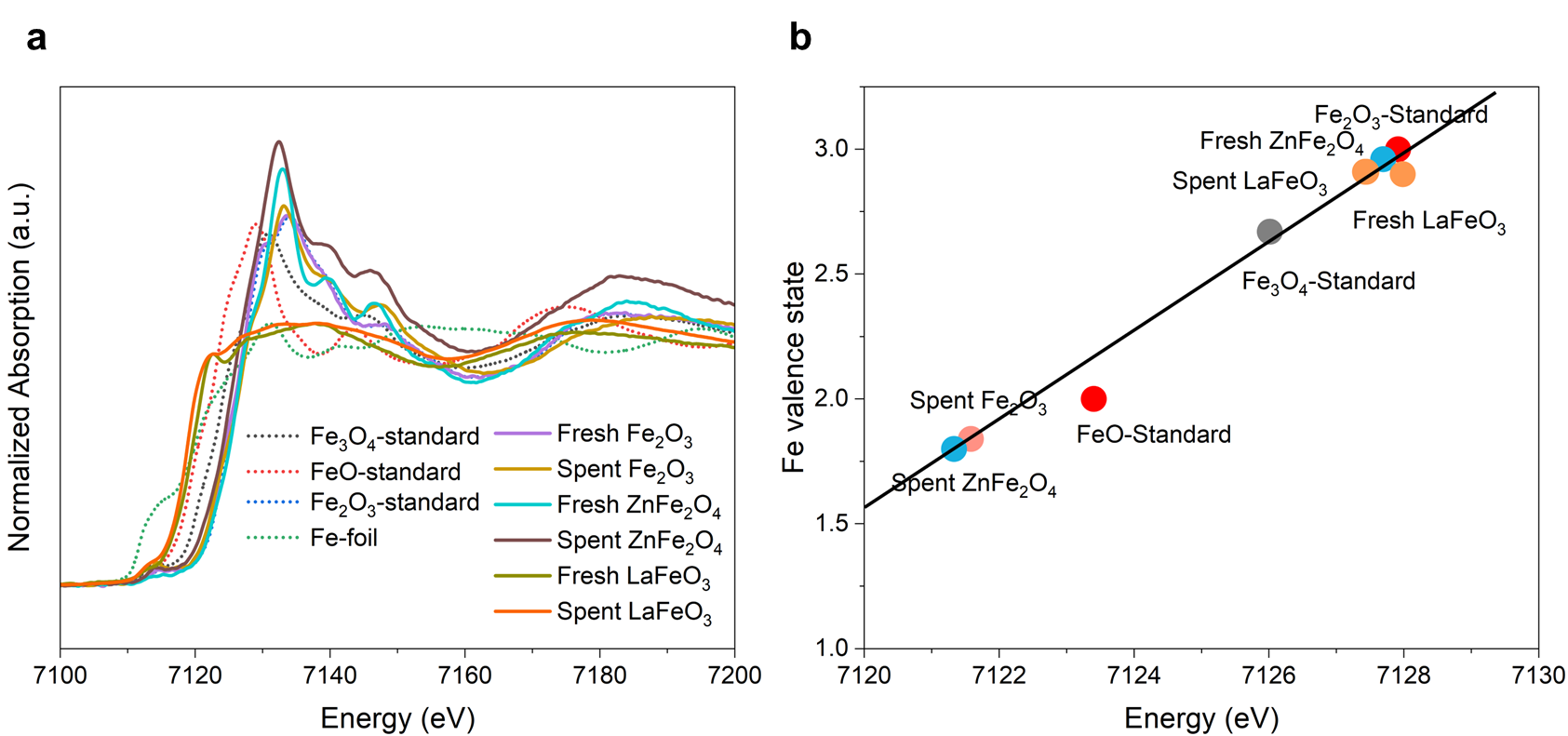


**Supplementary Fig. 18.** **EXANES spectra of Fe samples.** (**a**) Normalized Fe K-edge X-ray absorption near-edge structures. (**b**) The mean chemical valence of Fe species obtained by XANES.

**Note:** According to the normalized XANEs at the Fe-K edge, the spent Fe_2_O_3_ and ZnFe_2_O_4_ shift to lower energy compared to fresh samples, suggesting a reduced oxidation state of Fe after reaction. In contrast, the absorption edges of LaFeO_3_ remain nearly unchanged after reaction. The mean chemical valences of Fe species were estimated based on XANESs. As shown in **Supplementary Fig. 18**, the mean chemical valences of Fe in fresh and spent LaFeO_3_ are +2.94 and +2.90, respectively. These numbers decrease to 1.87 and 1.75 for spent Fe_2_O_3_ and ZnFe_2_O_4_, respectively. There must exist Fe species with chemical valences lower than +2 in the spent normal and spinel Fe oxides, which are deduced to be Fe carbides.


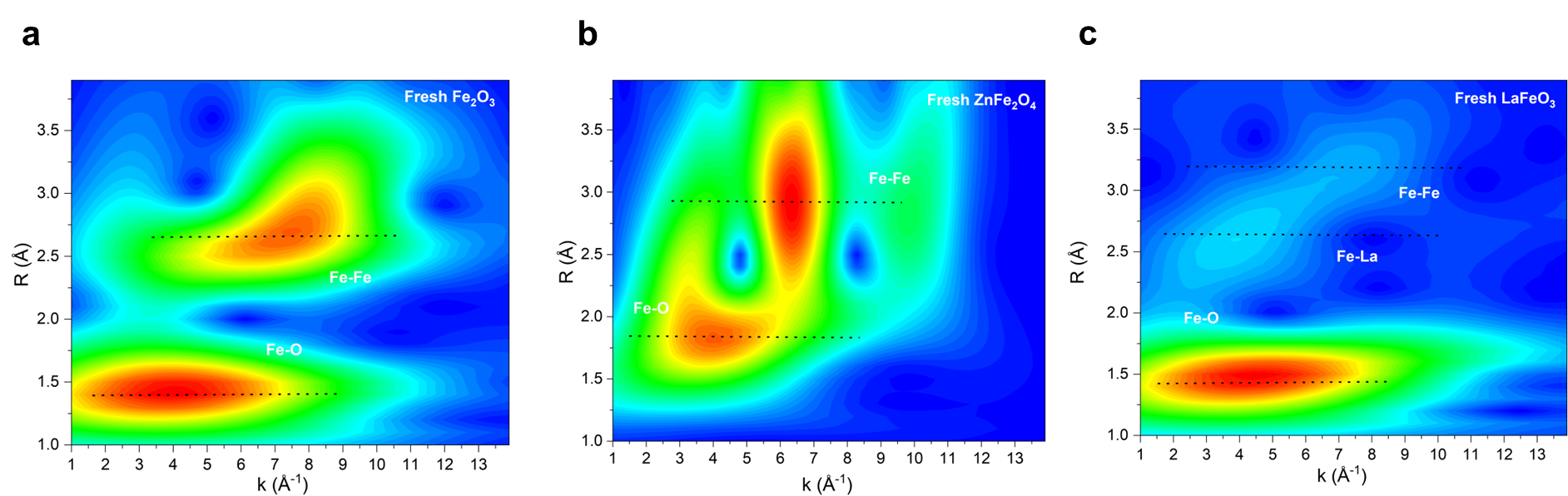


**Supplementary Fig. 19. Fe K-edge WT-EXAFS.** (a) Fe_2_O_3_, (b) ZnFe_2_O_4_, and (c) LaFeO_3_ catalysts.


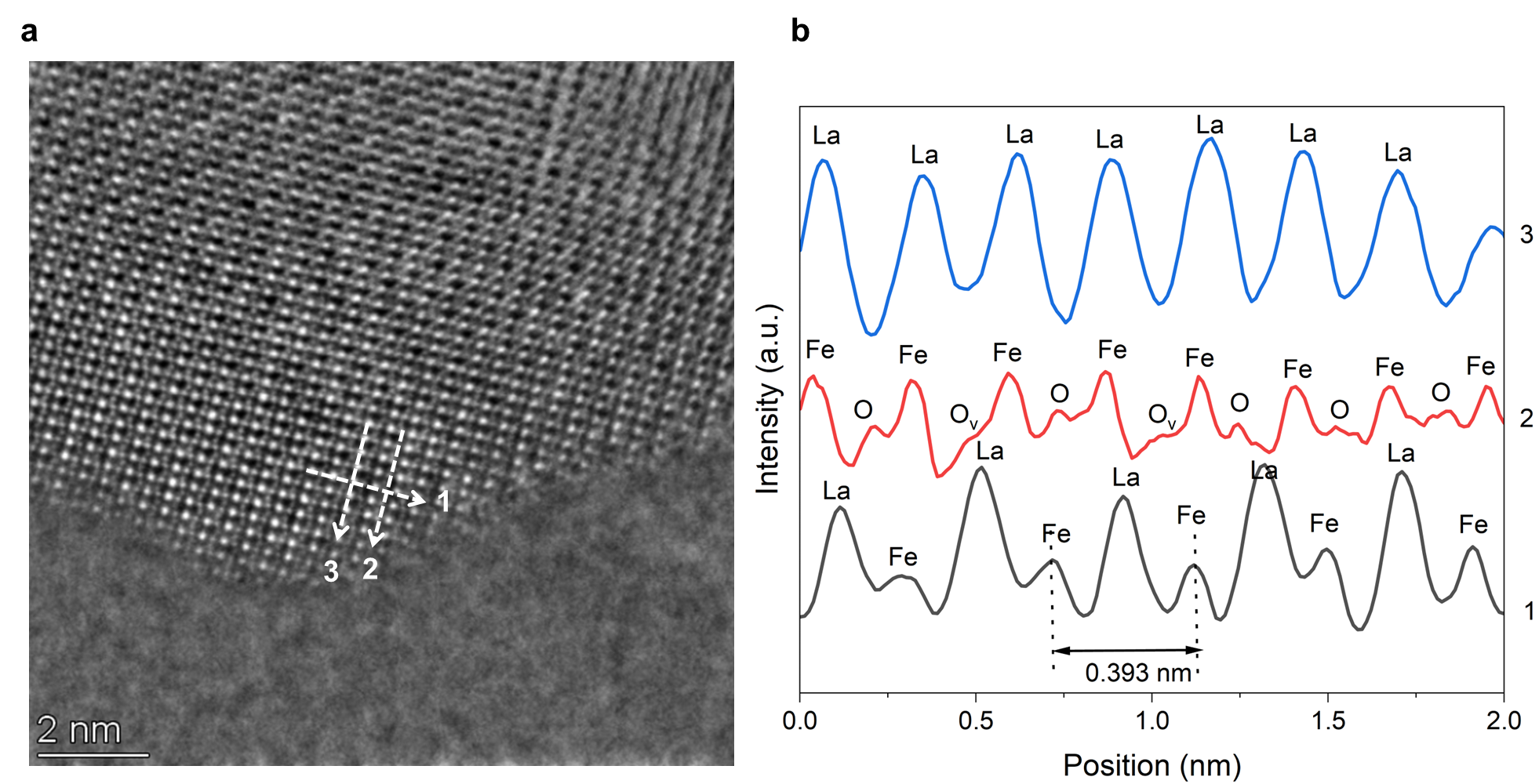


**Supplementary Fig. 20. Characterization of spent LaFe.** (a) iDPC-STEM image of spent LaFeO_3_ and (b) intensity profiles along the corresponding dashed lines in **a**.


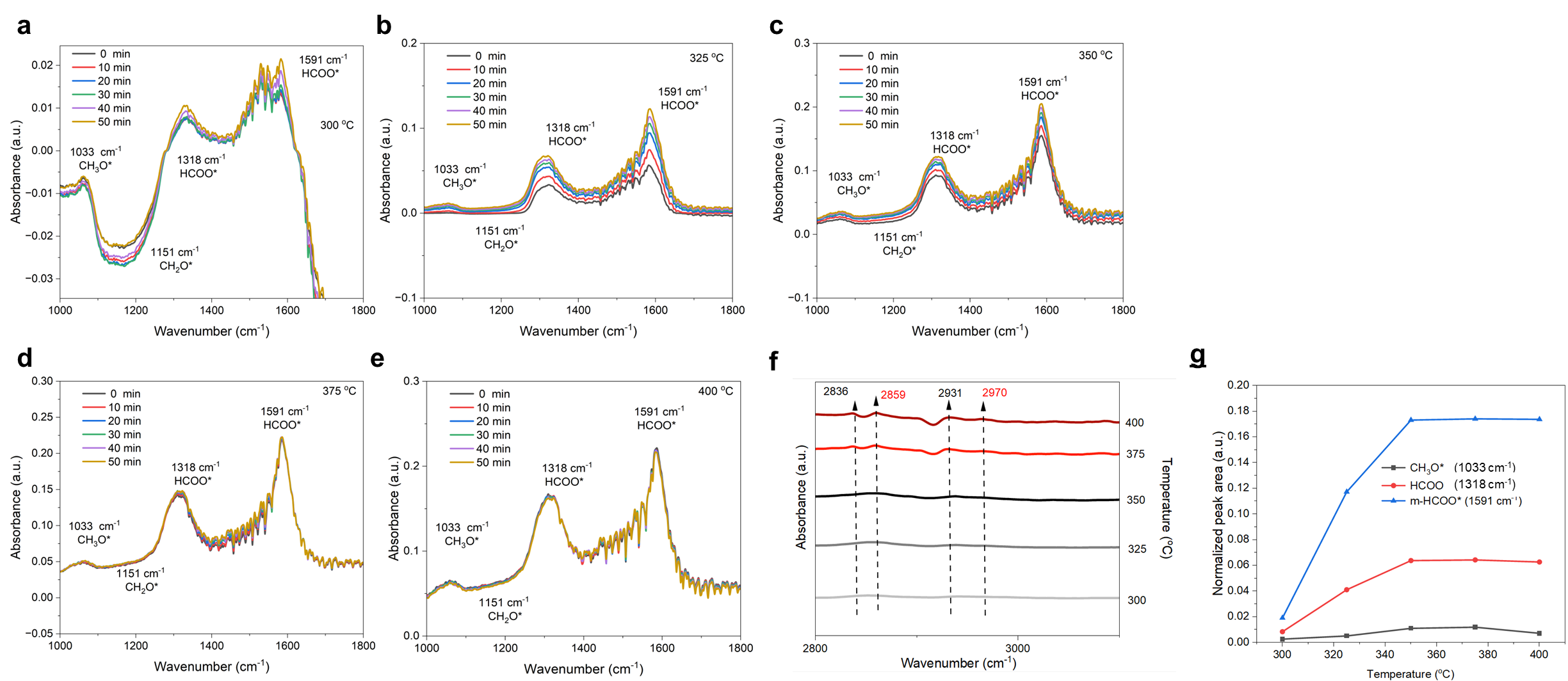


**Supplementary Fig. 21. In-situ temperature-programmed DRIFTS of LaFeO_3_.** DRIFTS spectra collected at (a) 300^o^C, (b) 325^o^C, (c) 350^o^C, (d) 375^o^C, and (e) 400^o^C. Each temperature was kept for 50 min. (f) The different temperature at the range of 2800-3200 cm^-1^ (g) The integrated peak area of signals for methoxy (1033 cm^−1^) and formate (1398 cm^−1^ and 1591 cm^−1^) as a function of temperature.

­­­­­­_­­_


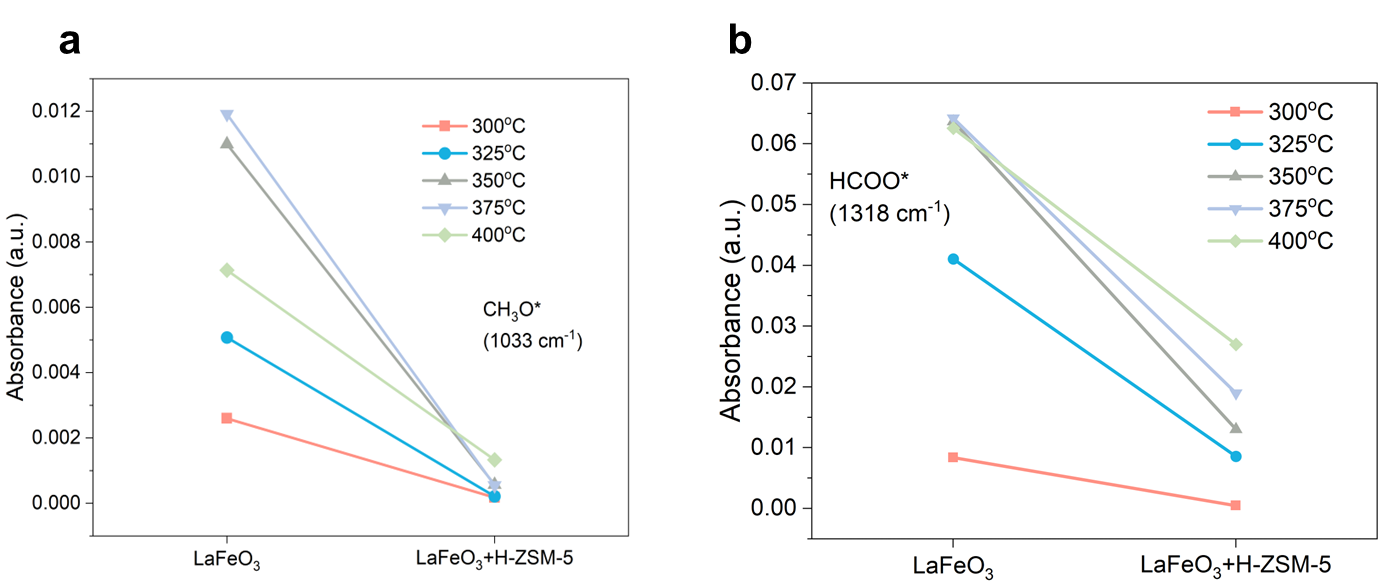


**Supplementary Fig. 22. Comparison between LaFeO_3_ and LaFeO_3_/H-ZSM-5, in terms of the integrated peak areas.** (a) methoxy (1033 cm^−1^) and (b) formate (1318 cm^−1^) at varying temperatures.


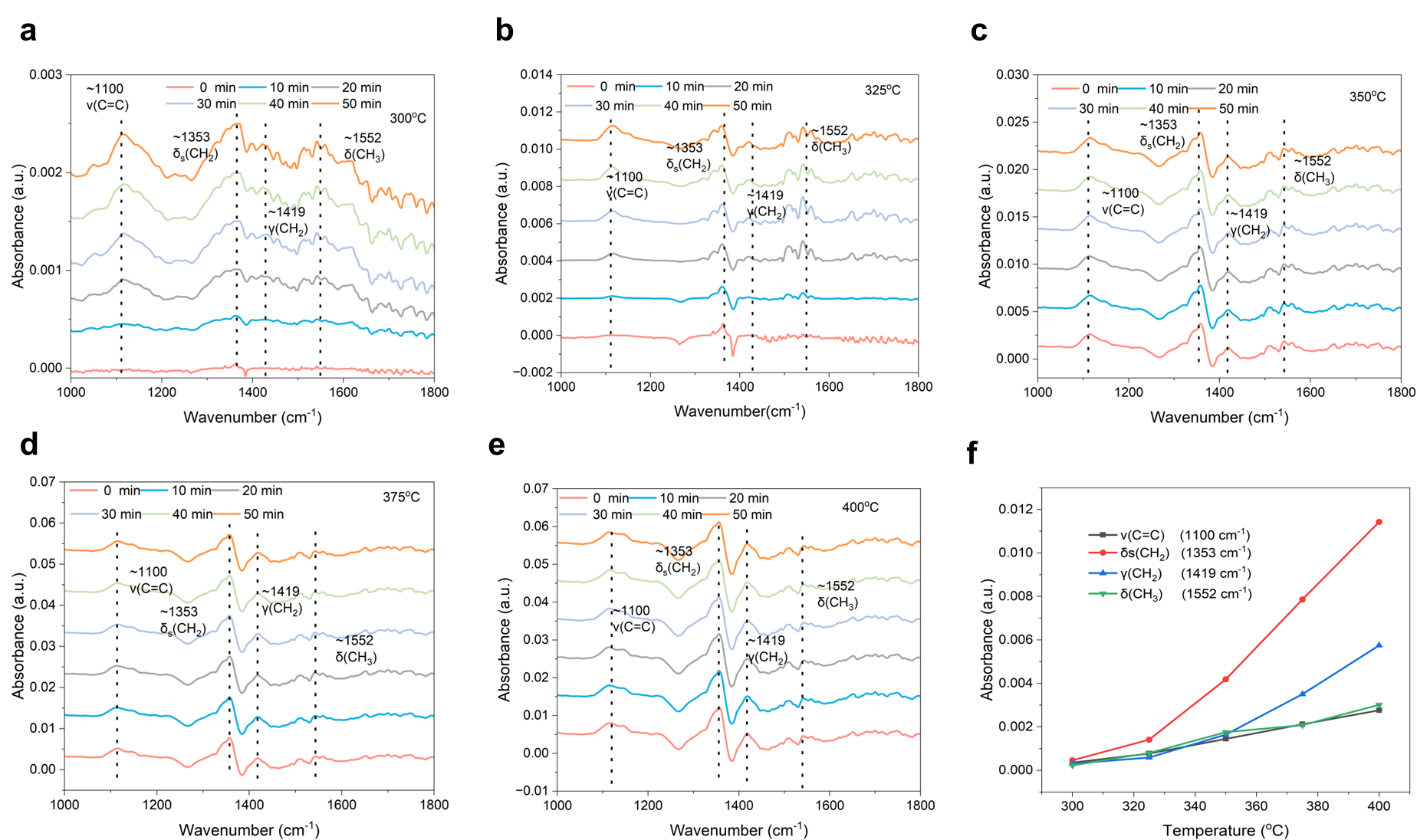


**Supplementary Fig. 23. In-situ temperature-programmed DRIFTS of Fe_x_C_y_ (derived from fresh Fe_2_O_3_).** DRIFTS spectra collected at (a) 300^o^C, (b) 325^o^C, (c) 350^o^C, (d) 375^o^C, and (e) 400^o^C. Each temperature was kept for 50 min. (f) The integrated peak area of signals for C=C (1100 cm^−1^), CH_2_ (1353 cm^−1^ and 1419 cm^−1^), and CH_3_ (1552 cm^−1^) as a function of temperature.


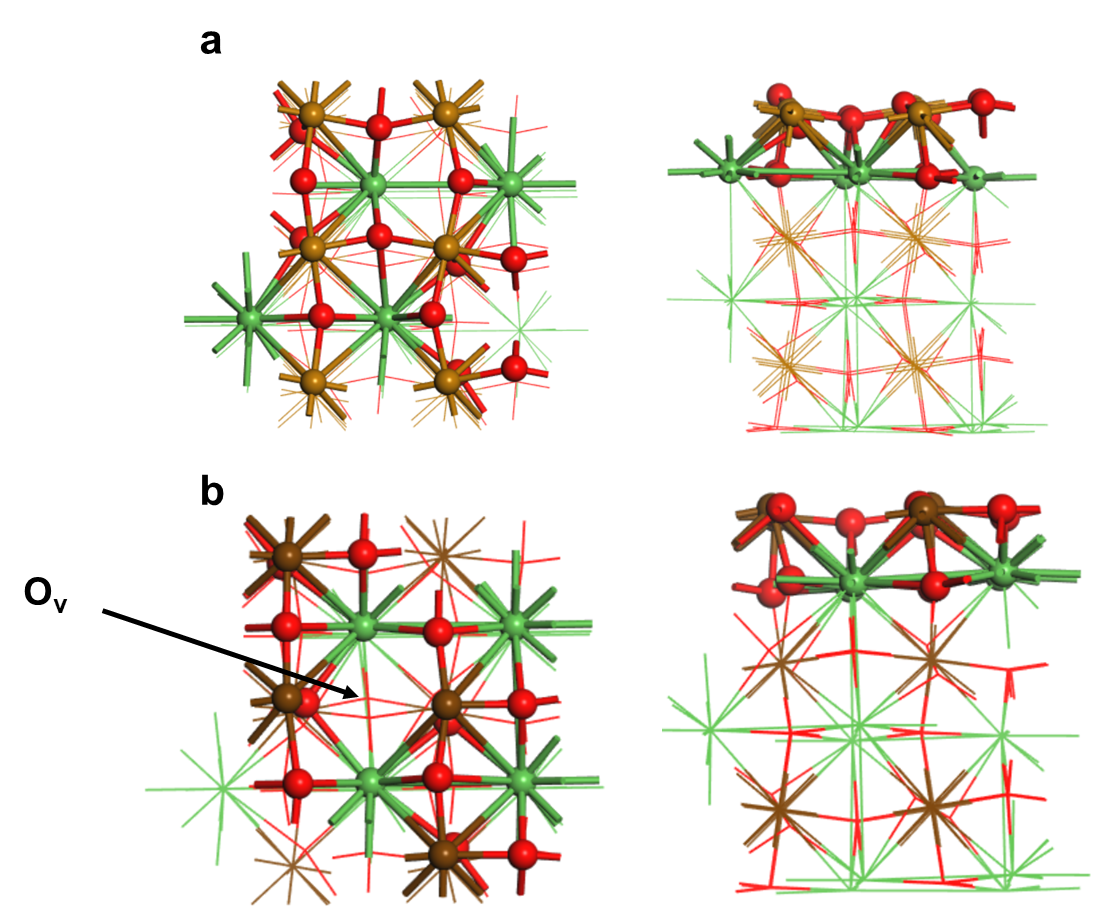


**Supplementary Fig. 24. Structure models of LaFeO_3_ (220) surface.** (a) without and (b) with O_v_ (green: La; brown: Fe; red: O). The concentration of O_v_ is 1/8 monolayer of surface O atoms.


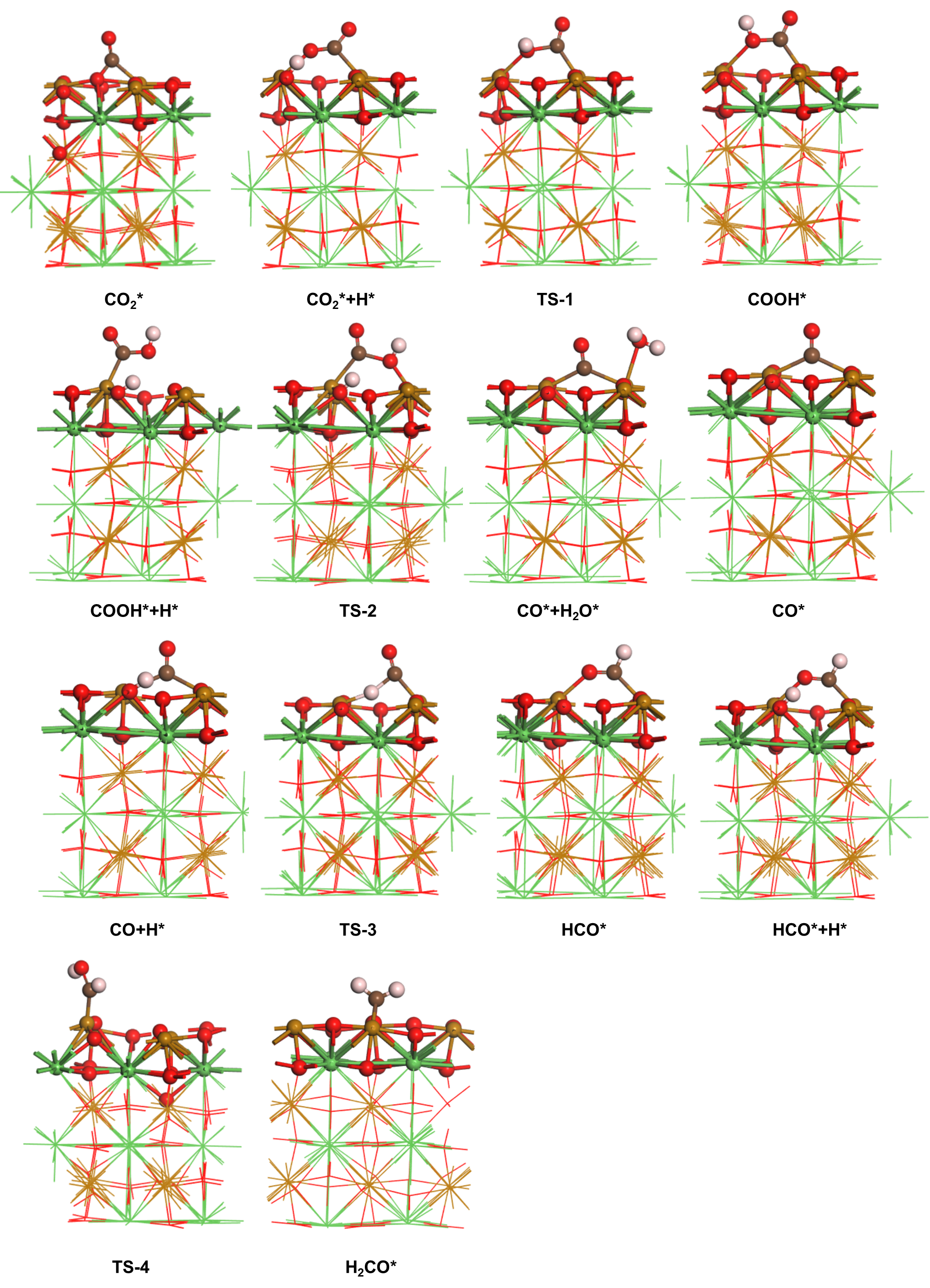


**Supplementary Fig. 25.** **Optimized atomic structures of surface species/states involved in the carboxyl-CO pathway of CO_2_ hydrogenation** (green: La; brown: Fe; red: O; grey: C; pink: H). The surface is LaFeO_3_ (220) with O_v_.


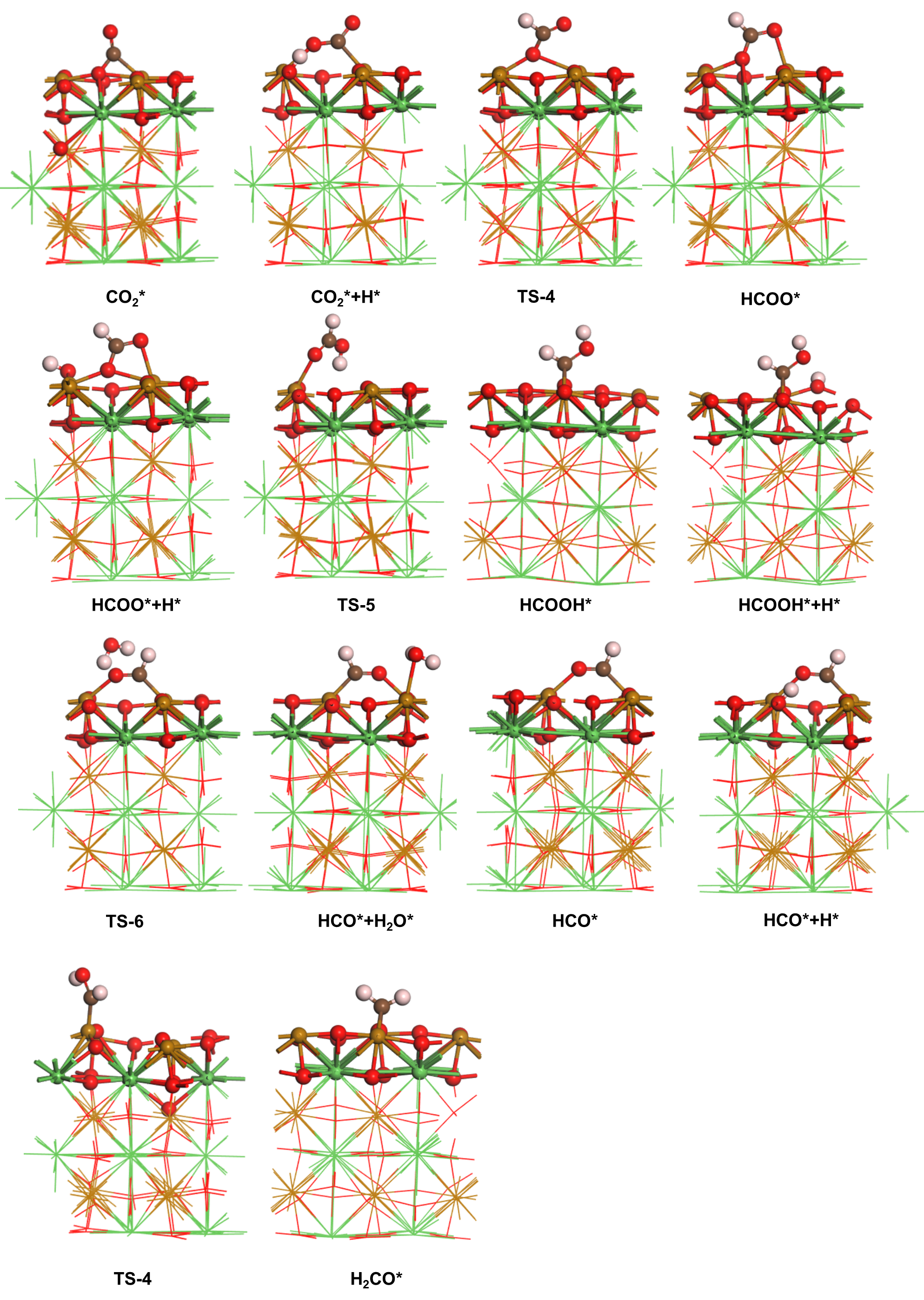


**Supplementary Fig. 26. Optimized atomic structures of surface species/states involved in the formate-HCOOH pathway of CO_2_ hydrogenation** (green: La; brown: Fe; red: O; grey: C; pink: H). The surface is LaFeO_3_ (220) with O_v_.


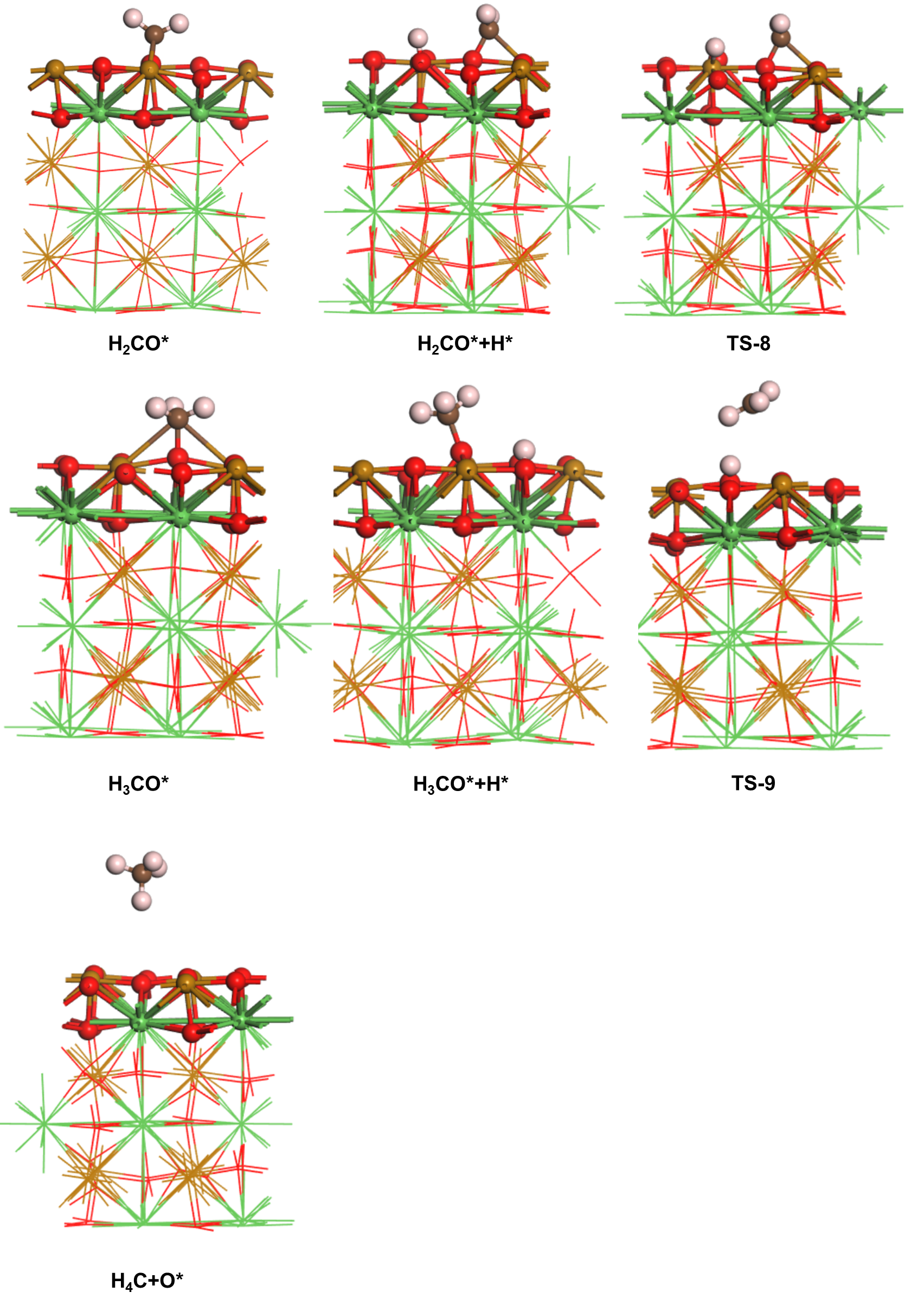


**Supplementary Fig. 27. Optimized atomic structures of surface species/states involved in the formaldehyde hydrogenation pathway of CO_2_ hydrogenation (green: La; brown: Fe; red: O; grey: C; pink: H).** The surface is LaFeO_3_ (220) with O_v_.


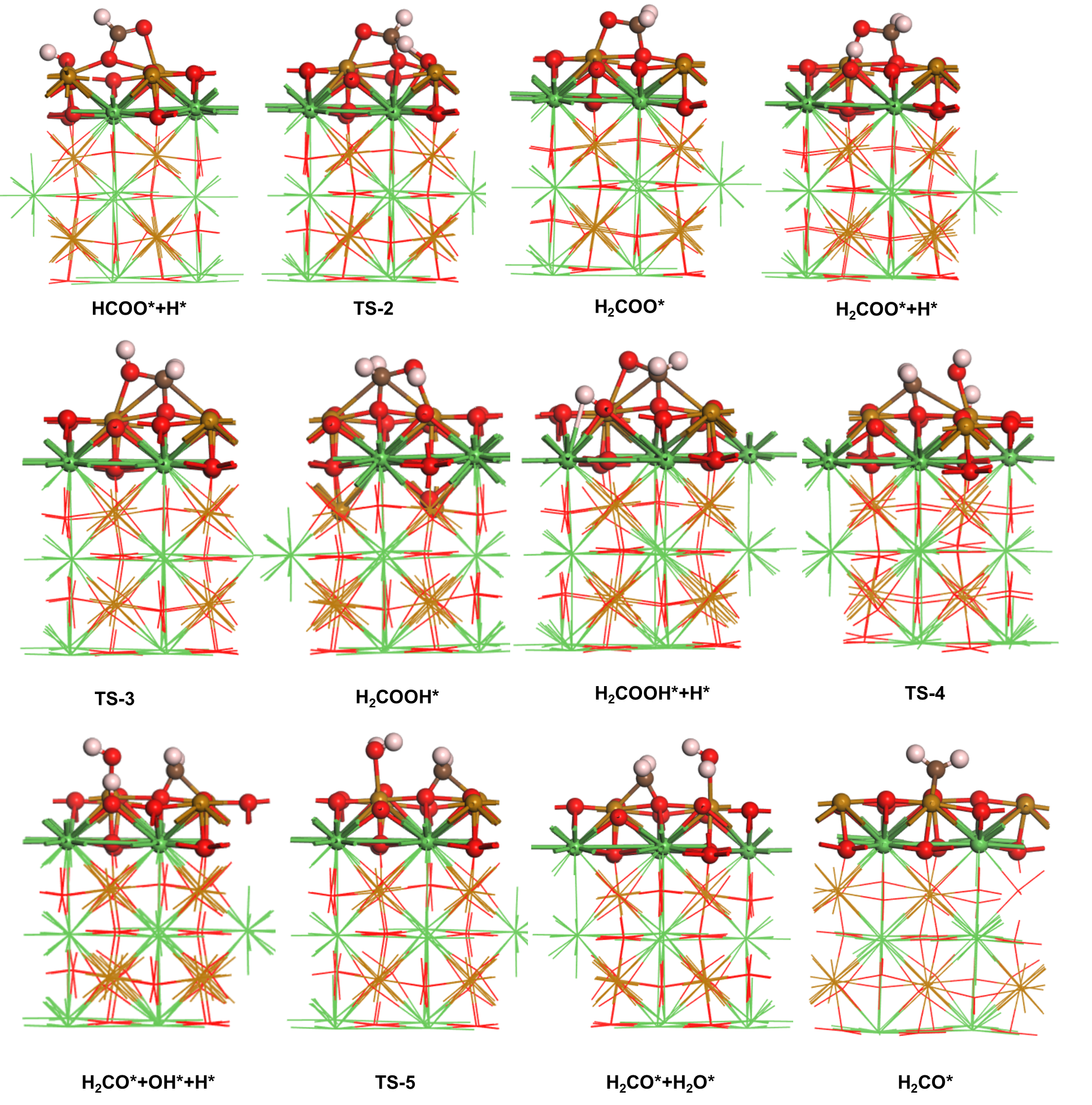


**Supplementary Fig. 28. Optimized atomic structures of surface species/states involved in the formate-H_2_COO pathway of CO_2_ hydrogenation** (green: La; brown: Fe; red: O; grey: C; pink: H). The surface is LaFeO_3_ (220) with O_v_.


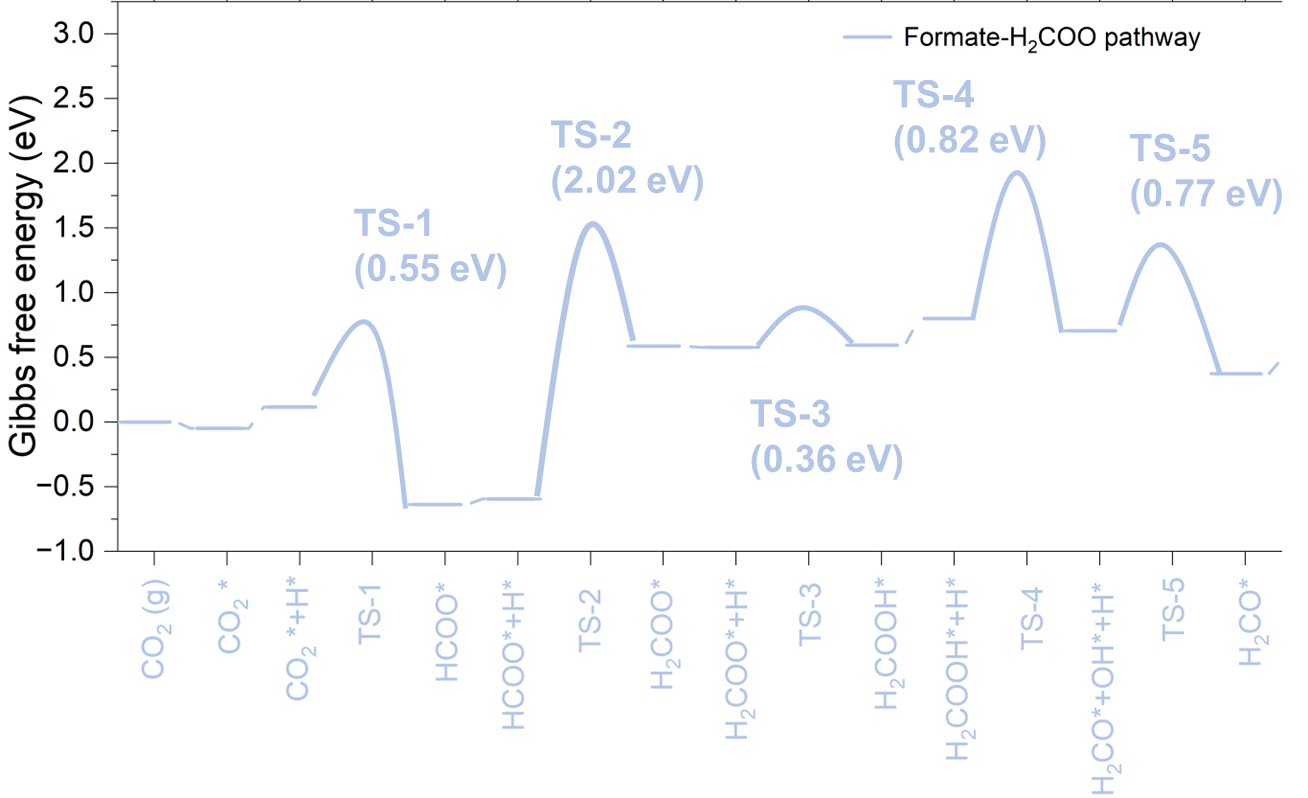


**Supplementary Fig. 29.** **Free energy diagram of an alternative pathway for CO_2_ hydrogenation initiating with formate.** The major difference lies in intermediates of H_2_COO* and H_2_COOH*.


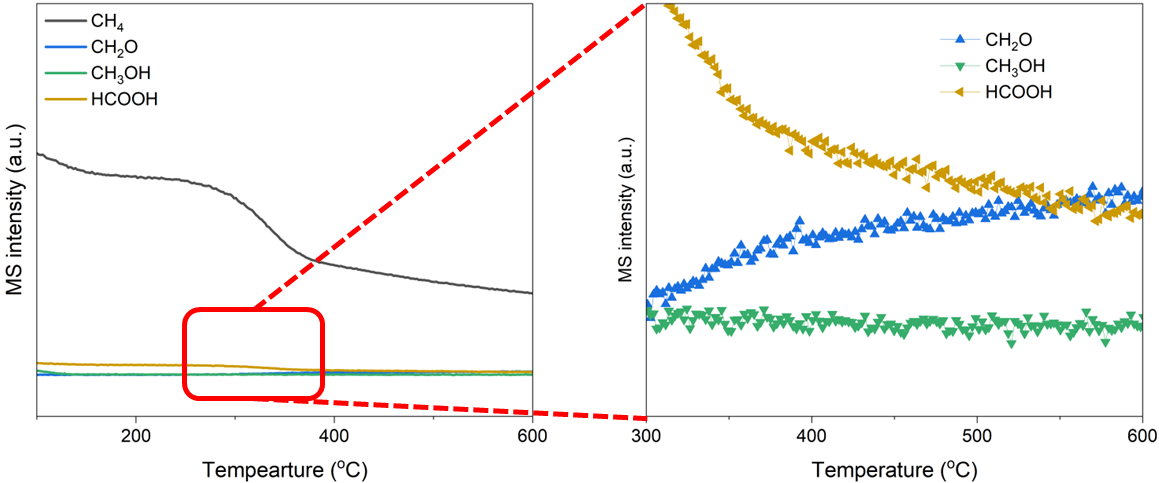


**Supplementary Fig. 30. Identification of intermediates.** Temperature-programmed surface reaction (TPSR) for identifying key intermediates of HCOOH (g) and H_2_CO (g) over LaFeO_3_ surface.


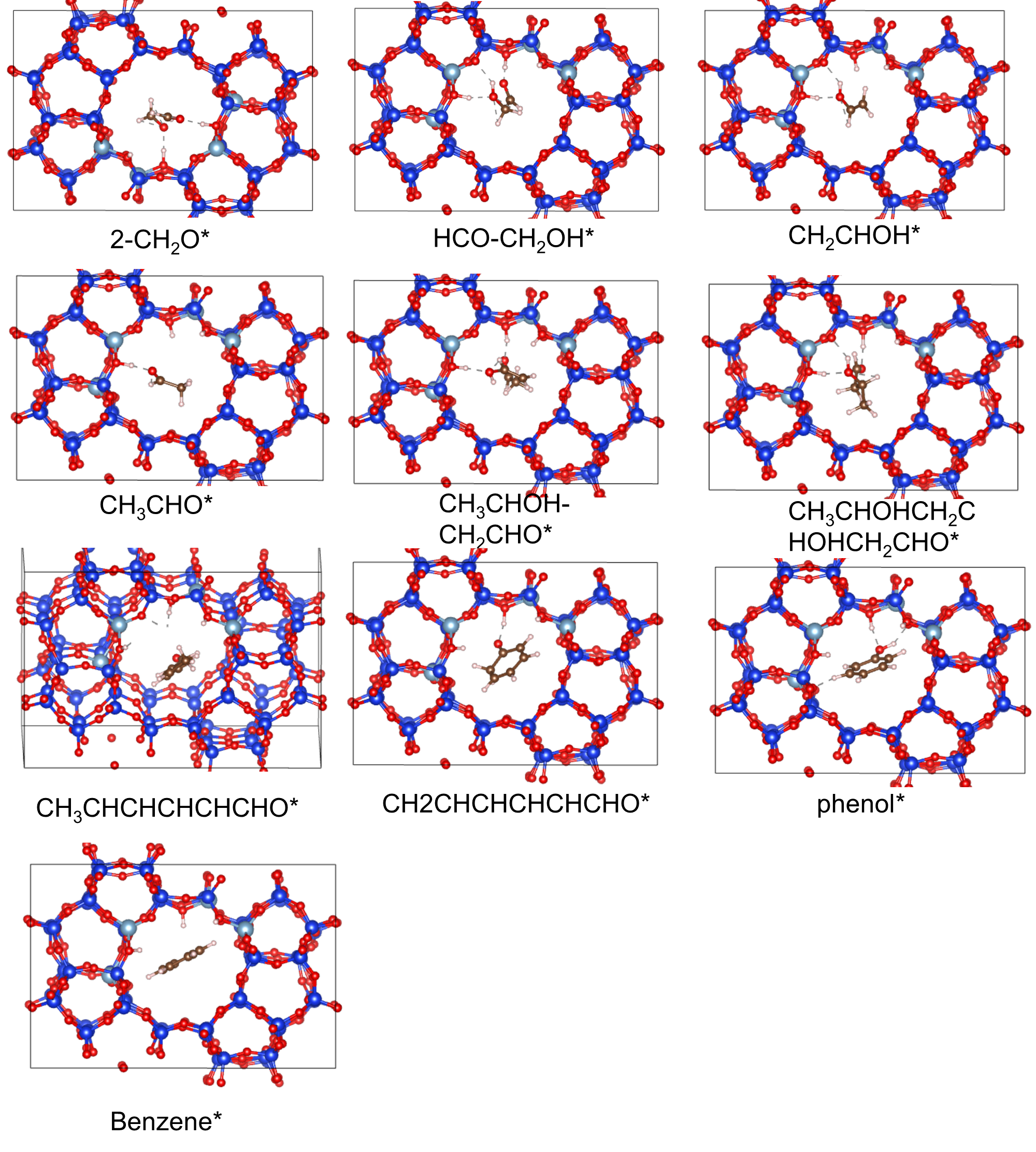


**Supplementary Fig. 31. Reaction pathway inside zeolites.** H_2_CO as a key intermediate inside zeolite to form aromatics. (Follow aldol-aromatic reaction pathway)

**3. Supplementary Tables and Notes**

**Supplementary Table 1.** Comparison between different composite catalytic systems for CO_2_-to-aromatics conversion

| Catalyst | Ref. | T / ^o^C | P / MPa | SV /  (ml h^−1^ g_cat_^−1^) | X(CO_2_) / % | S(Aromatic)  / % | | Aromatic  yield, % |  |
| --- | --- | --- | --- | --- | --- | --- | --- | --- | --- |
|  |  |  |  |  |  | CO-excluded | CO-included |  |  |
| ZnO-ZrO_2_+H-ZSM-5 | | ^12^ | 320 | 4.0 | 600 | 14.0 | 73.0 | 40.9 | 5.72 |
| ae-ZnO-ZrO_2_+H-ZSM-5 | | ^13^ | 340 | 4.0 | 1800 | 16.0 | 76.0 | 45.6 | 7.29 |
| ZnCr_2_O_4_+H-ZSM-5 | | ^14^ | 300 | 3.0 | 300 | 11.3 | 75.4 | 15.1 | 1.71 |
| ZnZrO_x_+chain-H-ZSM-5  Cr_2_O_3_+H-ZSM-5  Na-ZnFeO_x_+H-ZSM-5  FeMnO_x_+H-ZSM-5 | | ^15^  ^16^  ^17^  ^18^ | 320  350  320  320 | 3.0  3.0  3.0  3.0 | 1020  1200  1000  1000 | 17.5  34.5  41.2  44.5 | 75.0  76.0  75.6  64.2 | 56.4  60.8  70.6  57.8 | 9.87  23.6  29.1  25.7 |
| Cu-Fe_2_O_3_+H-ZSM-5 | | ^19^ | 320 | 3.0 | 1000 | 57.3 | 56.6 | 50.9 | 29.1 |
| K-Zn-Fe+H-ZSM-5  CuFeO_2_/0.15M-H-ZSM-5  ZnCr2O4+H-ZSM-5 | | ^20^  ^21^  ^22^ | 320  320  350 | 3.0  3.0  4.0 | 1000  8100  1200 | 42.6  52.8  23.4 | 45.2  69.7  66.0 | 38.4  64.6  54.3 | 16.3  34.1  12.7 |
| LaFeO_3_+H-ZSM-5 | | This work | 350 | 3.0 | 1000 | 61.2 | 85.8 | 75.5 | 47.5 |

1. Abbreviations. T: Temperature; P: Pressure; SV: Space velocity; X (CO_2_): CO_2_ conversion rate; S(Aromatics): Aromatics selectivity;
2. The CO_2_/H_2_ ratio is 1: 3 for all conditions.

**Supplementary Table 2.** Fit results of Fe K-edge of EXAFS spectra of various catalysts

| Sample | Paths | R (Å) | CNs | σ^2^ (Å^2^) | R-factor |
| --- | --- | --- | --- | --- | --- |
| Fresh Fe_2_O_3_ | Fe-O  Fe-Fe | 1.98  2.94 | 5.70  4.40 | 0.004  0.003 | 0.015  0.015 |
| Spent Fe_2_O_3_ | Fe-C  Fe-Fe | 1.99  2.67 | 4.73  7.45 | 0.014  0.008 | 0.006  0.006 |
| Fresh  ZnFe_2_O_4_ | Fe-O  Fe-Fe | 2.05  3.02 | 5.32  5.21 | 0.009  0.008 | 0.004  0.003 |
| Spent  ZnFe_2_O_4_ | Fe-C  Fe-Fe | 1.94  2.74 | 3.36  7.29 | 0.006  0.010 | 0.008  0.009 |
| Fresh LaFeO_3_ | Fe-O  Fe-Fe | 2.20  3.91 | 5.39  4.69 | 0.007  0.002 | 0.018  0.018 |
| Spent LaFeO_3_ | Fe-O  Fe-Fe | 2.21  3.90 | 5.15  4.85 | 0.007  0.010 | 0.015  0.015 |

**Note:** CNs, coordination numbers; R, distance between absorber and backscatter atoms; σ^2^, Debye–Waller factor; In the fits of the Fe-edge EXAFS spectra of various Fe oxide samples, S_0_^2^=0.81 fixed by Fe foil.

All the fitting results of Fe K-edge were performed using the ARTEMIS module built in IFEFFIT package and were done for k^2^-weighted χ(k) functions with Hanning windows (dk=1 Å^-1^). For all the Fe oxide samples, the FT-EXAFS curves show a distinct Fe-O/C peak at the nearest neighbor shell, within the R range of 1-2.3 Å. And a new peak appeared at 2.8-3.9 Å assigned to the Fe-Fe path contribution, due to their different k value in the wavelet transform analysis. Therefore, a structure model including Fe-O/C paths was used to fit the EXAFS data of these four samples. During the fittings, each of the Debye-Waller factors, coordination numbers, interatomic distances were treated as adjustable parameters for Fe-O and Fe-Fe paths.

In particular, the CN for the Fe–Fe path increase from 5.70 to 7.45 after reaction over Fe_2_O_3_. As for ZnFe_2_O_4_, the CN for the Fe-Fe path increase from 5.21 to 7.45 after reaction. Given the similar Fe–Fe CNs (ca. 3–4) in Fe_2_O_3_ and Fe_3_O_4_, such an increase is attributed to the formation of Fe carbides with Fe–Fe CNs exceeding 7. These results suggest the dynamic evolution of normal Fe to Fe carbide mixtures under reactive atmospheres. Unlike normal Fe, the coordination environments of Fe in LaFeO_3_ remain basically unchanged.

**Supplementary Table 3 the assignment of the FTIR wavelengths of the surface species in Figs. 4 and Supplementary Figs. 22-24.**

| Surface species | Wavenumber (cm^-1^) | Assignment |
| --- | --- | --- |
| Bidentate formate | 2970  2859  1591 | δ(CH)+v_as_(O-C-O)  v(C-H)  v_as_(O-C-O) |
|  | 1384 | v_s_(O-C-O) |
|  | 1312 | δ(C-H) of H-COO |
| methoxy | 2931  2836  1033 | v_as_(CH_3_)  v_s_(CH_3_)  v(C-O) of CH_3_-O |
| Formaldehyde | 1750  1501 | v(C=O)  δ(CH_2_) |
| Aldol condensates/C-C coupling products | 1440  1200  1100 | CH_3_CHO*  v(C-C)  v(C=C) |

**Supplementary Table 4.** Adsorption Gibbs free energy (eV) of surface species/states involved in CO_2_ hydrogenation on LaFeO_3_ (220) surfaces without and with O_v_.

|  | without O_v_ | with O_v_ |  | without O_v_ | with O_v_ |
| --- | --- | --- | --- | --- | --- |
| CO_2_* | 1.11 | −0.05 | CO_2_*+H* | 0.99 | 0.12 |
| HCOO* | 1.89 | −0.64 | HCOO+H* | 1.51 | −0.59 |
| COOH* | 2.64 | 0.84 | COOH*+H* | 3.36 | 1.04 |
| HCOOH* | 2.33 | 0.76 | HCOOH*+H* | 1.86 | 0.49 |
| H_2_COO* | 3.34 | 0.59 | H_2_COO*+H* | 3.30 | 0.58 |
| H_2_COOH* | 3.12 | 0.59 | H_2_COOH*+H* | 2.85 | 0.80 |
| H_2_CO*+OH*+H* | 2.86 | 0.71 | H_2_CO*+H_2_O* | 2.95 | 0.37 |
| CO* | 1.18 | −0.06 | CO*+H* | 0.95 | 0.24 |
| HCO*+H_2_O* | 2.74 | 0.94 | CO*+H_2_O* | 2.19 | 0.77 |
| HCO* | 1.99 | 0.31 | HCO*+H* | 1.81 | 0.40 |
| H_2_CO* | 1.98 | 0.50 | H_2_CO*+H* | 1.87 | 0.58 |
| H_3_CO* | 2.21 | 0.52 | H_3_CO*+H* | 1.86 | 0.40 |
| H* | 0.46 | −0.78 | O* | 2.64 | −0.85 |

**Supplementary Table 5.** Reaction thermodynamics of aromatics formation

| Reactant | Reaction energy to C_6_H_6_ (eV per C) | Reaction energy to C_10_H_14_ (eV per C) |
| --- | --- | --- |
| CO_2_ | −0.51 | −0.50 |
| HCOOH | −1.23 | −1.22 |
| H_2_CO | −0.88 | −0.87 |

**4. Supplementary References**

1 Kresse, G. & Furthmüller, J. Efficient iterative schemes for ab initio total-energy calculations using a plane-wave basis set. *Physical review B* **54**, 11169 (1996).

2 Perdew, J. P., Burke, K. & Ernzerhof, M. Generalized gradient approximation made simple. *Physical review letters* **77**, 3865 (1996).

3 Wang, X. *et al.* eg occupancy as an effective descriptor for the catalytic activity of perovskite oxide-based peroxidase mimics. *Nature Communications* **10**, 704 (2019).

4 Yun, T. G., Heo, Y., Bin Bae, H. & Chung, S.-Y. Elucidating intrinsic contribution of d-orbital states to oxygen evolution electrocatalysis in oxides. *Nature Communications* **12**, 824 (2021).

5 Ye, X. *et al.* Observation of novel charge ordering and spin reorientation in perovskite oxide PbFeO3. *Nature communications* **12**, 1917 (2021).

6 Zhang, X. *et al.* FeO6 octahedral distortion activates lattice oxygen in perovskite ferrite for methane partial oxidation coupled with CO2 splitting. *Journal of the American Chemical Society* **142**, 11540-11549 (2020).

7 Kresse, G. & Joubert, D. From ultrasoft pseudopotentials to the projector augmented-wave method. *Physical review b* **59**, 1758 (1999).

8 Monkhorst, H. J. & Pack, J. D. Special points for Brillouin-zone integrations. *Physical review B* **13**, 5188 (1976).

9 Christensen, R., Hansen, H. A. & Vegge, T. Identifying systematic DFT errors in catalytic reactions. *Catalysis Science & Technology* **5**, 4946-4949 (2015).

10 Granda-Marulanda, L. P. *et al.* A semiempirical method to detect and correct DFT-based gas-phase errors and its application in electrocatalysis. *Acs Catalysis* **10**, 6900-6907 (2020).

11 Liu, Q.-Y., Shang, C. & Liu, Z.-P. In situ active site for CO activation in Fe-catalyzed Fischer–Tropsch synthesis from machine learning. *Journal of the American Chemical Society* **143**, 11109-11120 (2021).

12 Li, Z. *et al.* Highly selective conversion of carbon dioxide to aromatics over tandem catalysts. *Joule* **3**, 570-583 (2019).

13 Zhou, C. *et al.* Highly active ZnO-ZrO2 aerogels integrated with H-ZSM-5 for aromatics synthesis from carbon dioxide. *ACs Catalysis* **10**, 302-310 (2019).

14 Arslan, M. T. *et al.* Highly selective conversion of CO2 or CO into precursors for kerosene-based aviation fuel via an aldol–aromatic mechanism. *ACS Catalysis* **12**, 2023-2033 (2022).

15 Wang, T. *et al.* ZnZrOx integrated with chain-like nanocrystal HZSM-5 as efficient catalysts for aromatics synthesis from CO2 hydrogenation. *Applied Catalysis B: Environmental* **286**, 119929 (2021).

16 Wang, Y. *et al.* Rationally designing bifunctional catalysts as an efficient strategy to boost CO2 hydrogenation producing value-added aromatics. *Acs Catalysis* **9**, 895-901 (2018).

17 Jiang, Q. *et al.* Selective Hydrogenation of CO2 to Aromatics over Composite Catalyst Comprising NaZnFe and Polyethylene Glycol-Modified HZSM-5 with Intra-and Intercrystalline Mesoporous Structure. *Industrial & Engineering Chemistry Research* (2023).

18 Song, G. *et al.* Tuning the Integration Proximity between Na Promoter and FeMnO x Coupled with Rationally Modified HZSM-5 to Promote Selective CO2 Hydrogenation to Aromatics. *Industrial & Engineering Chemistry Research* **61**, 6820-6830 (2022).

19 Song, G., Li, M., Yan, P., Nawaz, M. A. & Liu, D. High conversion to aromatics via CO2-FT over a CO-reduced Cu-Fe2O3 catalyst integrated with HZSM-5. *ACS Catalysis* **10**, 11268-11279 (2020).

20 Liang, J. *et al.* Direct conversion of CO2 to aromatics over K–Zn–Fe/ZSM-5 catalysts via a Fischer–Tropsch synthesis pathway. *Industrial & Engineering Chemistry Research* **61**, 10336-10346 (2022).

21 Cheng, Y. *et al.* High-yield production of aromatics over CuFeO 2/hierarchical HZSM-5 via CO 2 Fischer–Tropsch synthesis. *Green Chemistry* **25**, 3570-3584 (2023).

22 Gao, W. *et al.* Selective Conversion of CO2 into para‐Xylene over a ZnCr2O4‐ZSM‐5 Catalyst. *ChemSusChem* **13**, 6541-6545 (2020).
